# Live biotherapeutic *enterococcus lactis* MNC-168 promotes the efficacy of immune checkpoint blockade in cancer therapy by activating STING pathway via bacterial membrane vesicles

**DOI:** 10.1080/19490976.2025.2557978

**Published:** 2025-09-12

**Authors:** Yibo Xian, Zhipeng Chen, Zhou Lan, Chenchen Zhang, Hao Sun, Zhenzhen Liu, Ping Kong, Yajun Liang, Yingying Zhao, Si-Yang Maggie Liu, Yiqi Zhou, Linchuan Gan, Baoxia Li, Xue Su, Baojia Huang, Chen Xiao, Ruijuan Zhu, Guozhen Zhao, Canshan Lao, Chuan-Sheng Lin, Dongya Zhang, Xianzhi Jiang

**Affiliations:** a Moon (Guangzhou) Biotech Co. Ltd, Guangdong, China; b Guangdong Lung Cancer Institute, Guangdong Provincial People’s Hospital (Guangdong Academy of Medical Sciences), Southern Medical University, Guangzhou, China

**Keywords:** *Enterococcus lactis*, MNC-168, immune system, immunotherapy, STING, bacterial membrane vesicles

## Abstract

The gut microbiome has the potential to influence tumor development and affect the efficacy of cancer therapeutics, particularly immunotherapy. However, the specific species and strains rather than all microbes that promote antitumor immunity by modulating the function of systemic immunity or tumor-infiltrating lymphocytes (TILs) in tumor environments remain to be elucidated. In this study, we analyzed the microbiome composition of responders and non-responders to PD-1 blockade therapy from a clinical cohort and found that *Enterococcus spp*. were abundant in the responders. Through in vitro screening, we identified Enterococcus lactis MNC-168, a commensal bacterium isolated from a healthy individual, which significantly inhibited tumor growth and enhanced the efficacy of anti-PD-1 treatment by promoting antitumor immunity. Mechanistically, MNC-168 activates innate immunity through a STING-IFN-I (stimulator of interferon genes-type I interferons) dependent pathway by releasing bacterial membrane vesicles (MVs), and targeting tumor tissue, thereby augmenting the antitumor immune response. Furthermore, we have confirmed the safety profile of MNC-168 and its enhancing effect on Anti-PD-1 activity across multiple preclinical models, as well as its potential clinical relevance to Anti-PD-1 therapy. These findings suggest that MNC-168 could represent a promising strategy for cancer therapy and has the potential to improve the efficacy of current immunotherapies.

## Introduction

Mammals harbor widely diverse and extremely active microbial communities, known as microbiota, with the gastrointestinal (GI) tract serving as their primary habitat.^1^ The gut microbiota has undergone co-evolution with its host over millennia, providing benefits such as digestion, nutrient production, detoxification, protection against pathogens and regulation of the immune system.^1^ Recently, several approaches have demonstrated that the critical role of gut microbiota in the development and maintenance of immune system homeostasis, such as using the germ-free (GF) models.^2^ and microbiota manipulation, either with antibiotic treatment or microbiota reconstitution.^3,4^ Notably, gut microbiota can regulate not only the local intestinal immune system but also exert a profound influence on systemic immune responses through their metabolites, including toxins, pathogen-associated molecular patterns (PAMPs), and short-chain fatty acids (SCFA).^5,6^

In recent years, the emergence of cancer immunotherapy has revolutionized current cancer treatments,^7^ with the PD-L1/PD-1 blockade serving as a pivotal cornerstone.^8^ However, the limited efficacy of current immunotherapies in most patients can be attributed to intrinsic and extrinsic factors of tumor cells, including primary, adaptive, and acquired resistance to immunotherapy, as well as the presence of an immunosuppressive microenvironment.^9^ Emerging evidence indicates that microbes are important regulators of innate and adaptive immunity,^10^ with the potential to modulate responses to not only chemotherapeutic reagents.^11^ but also immunotherapeutic agents for cancer therapy.^12,13^ In preclinical studies, several specific species and strains of microbes have been identified that improve the efficacy of chemotherapy or immunotherapy for cancer by modulating the tumor microenvironment, such as *Bifidobacterium bifidum*,^14^ *Lactococcus lactis*,^15^ and *Akkermansia muciniphila* (*A. muciniphila*).^16^ In clinical settings, the combination of a live biotherapeutic drug CBM588 (*Clostridium butyricum*) with nivolumab plus ipilimuma dramatically extended – progression-free survival (PFS) in patients with metastatic renal cell carcinoma (mRCC) from 2.5 months to 12.7 months,^17^ which shows the powerful potential of LBP in cancer treatment.

Recently, several molecular mechanisms underlying the enhancement of immunotherapy by the gut microbiota have been elucidated. For instance, Griffin et al. revealed that *Enterococcus* peptidoglycan remodeling promotes immune checkpoint inhibitors (ICIs) immunotherapy via NOD2 signaling.^18^ Bender et al. demonstrated that *Lactobacillus reuteri* could enhance ICIs efficacy in antitumor immunity through the AhR pathway by producing indole-3-aldehyde (I3A).^19^ The PAMPs, cGAS-STING pathway, which can be activated by bacterial cyclic dinucleotides (CDNs) or bacterial membrane vesicles (MVs), plays a pivotal role not only in pathogen defense but also in anti-tumor immunity.^20^ Lam et al. uncovered that *A. muciniphila*-derived STING agonists (e.g., cdAMP) induced IFN-I production by intra-tumoral mononuclear phagocytes to skew the TME and enhance ICIs efficacy,^21^ while Si et al. also demonstrated that *Lactobacillus rhamnosus GG* induced cGAS/STING-type I interferon and improved response to immune checkpoint blockade.^22^ Despite the consensus regarding the beneficial role of microbes in cancer therapies, recent reports have highlighted the positive influence of specific microbiomes on cancer.^23–25^ Therefore, the specific functions and molecular mechanisms of each component of the complex gut microbiome in cancer progression and therapy remain to be elucidated.

In this study, we compared the composition of microbiomes between responders (R) and non-responders (NR) to anti-PD-1 treatment in melanoma, non-small cell lung cancer, and renal cell carcinoma (NSCLC/RCC) patients and found that *Enterococcus spp*. were significantly enriched in the responders. Using an *in vitro* screening model, we identified that a specific *Enterococcus lactis* MNC-168 (MNC-168) strain isolated from a healthy individual significantly inhibited MC38 growth and enhanced the efficacy of anti-PD-1 treatment by eliciting a systemic immune response and enhancing T cell anti-tumor immunity. Mechanistically, we elucidated that MNC-168 released MVs could extravasate and preferentially target the tumor site via systemic circulation to trigger the STING pathway to contribute to antitumor immunity. More importantly, we validated the augmenting effect of MNC-168 on anti-PD-1 in multiple preclinical cancer models and demonstrated the potential of *Enterococcus lactis* to augment the anti-PD-1 response in clinical therapy. These findings suggest that MNC-168 has the potential to serve as a live biotherapeutic agent to augment cancer therapy.

## Results

### Enterococcus lactis MNC-168 inhibits tumor growth and enhances anti-PD-1 therapy

To identify the specific microbial signatures related to the response to cancer anti-PD-1 therapy, we collected publicly available fecal metagenomic sequencing (MGS) data from studies on the gut microbiome effects of cancer anti-PD-1 therapy.^16,26^ and analyzed the microbial compositional differences between responders (R) and non-responders (NR). We found that *Lactobacillus*, *Lactococcus*, and *Enterococcus* were enriched in the R group in both melanoma and NSCLC/RCC studies (Fig S1, supplementary file 1–2), which have been reported to prevent tumor development or facilitate antitumor immunity.^27–29^ Notably, the role of *Enterococcus spp*. in cancer remains controversial, which may be due to specific strains, experimental conditions, or the situation of cancer.^30^ Although commensal strains of these bacteria have been used as probiotics in animals and humans,^31^ antibiotic-resistant strains of *Enterococcus from* clinic can be pathogenic.^32^ For instance, *Enterococcus faecalis* and *Enterococcus faecium* are the predominant pathogenic species encountered in clinical settings, responsible for a range of infections and contributing to drug resistance in patients.^33,34^ To assess the potential risks of our strains isolated from health individual, we firstly compared the genome similarity, virulence factors, and resistance genes between our isolated strains and pathogenic strains isolated from clinical settings. Our results revealed pronounced genetic distinctions between the two groups of strains, characterized by substantially reduced levels of virulence factors and resistance genes in our strains compared to the clinical settings (Fig. S2a-2c).

**Figure 1.**
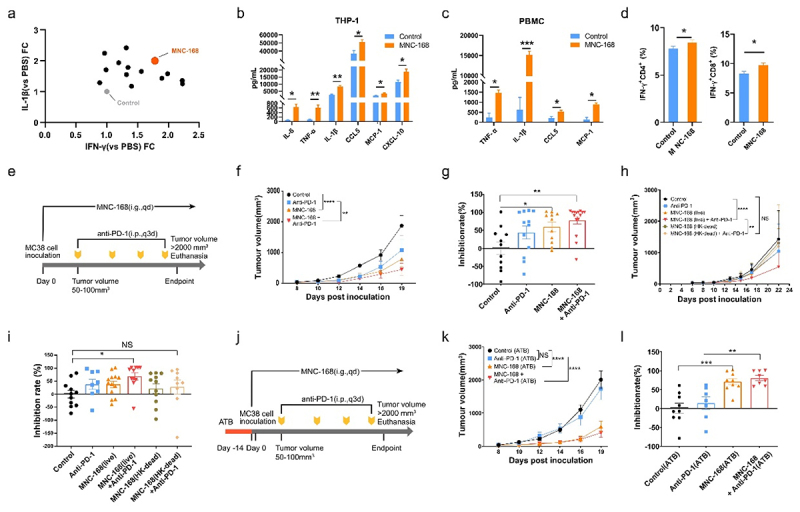
MNC-168 inhibits tumor growth and enhances anti-PD-1 therapy.

To determine whether *Enterococcus* influences antitumor immunity, we compared interferon-γ (IFN-γ) and interleukin 1 beta (IL-1β) production elicited by five genera of *Enterococcus* species isolated from healthy human feces using human peripheral blood mononuclear cells (PBMCs) and THP-1 *in vitro* co-culture models. We found that most of these strains significantly elicited IFN-γ or IL-1β secretion in both models (Supplementary file 3). However, *Enterococcus lactis* MNC-168 significantly increased IFN-γ and IL-1β production in both the *in vitro* models (Figure 1(a) and Supplementary file 3). Therefore, we focused on evaluating the chemokines and cytokines with cytometric beads array (CBA) to determine the influence of MNC-168 in both THP-1 and PBMC co-culture models. MNC-168 significantly increased the levels of pro-inflammatory factors, including IL-6, TNF-α, and IL-1β, as well as chemokines, such as CCL5, MCP-1, and CXCL10, in the THP-1 co-culture model (Figure 1(b)). Similarly, MNC-168 increased the levels of TNF-α, IL-1β, CCL5, and MCP-1 (Figure 1(c)) in the PBMC co-culture supernatants. Furthermore, in the PBMC co-culture model, the percentage of IFN-γ^+^CD4^+^ and IFN-γ^+^CD8^+^ T cells was increased by MNC-168 (Figure 1(d)), which was consistent with the increase in IFN-γ secretion (Figure 1(a)). *Enterococcus* was enriched in patients who responded to immunotherapy, which potentially increased immune activation.

a. Immune activated capacity of *Enterococcus spps*. was evaluated within human primary PBMCs and THP-1 co-culture *in vitro* models. IFN-γ and IL-1β, respectively, derived from PBMC and THP-1 were detected with ELISA. b. Cytometric Beads Array (CBA) of cytokines and chemokines in MNC-168 treated THP-1 model. MNC-168 (1 MOI) was co-cultured with THP-1 for 4 h before ATB was added, and the concentration of IL-6, TNF-α, IL-1β, CCL5, MCP-1, and CXCL10 within the supernatant was detected with CBA at 24 h after co-culture. c. CBA of MNC-168 treated PBMCs model. Concentration of TNF-α, IL-1β, CCL5 and MCP-1 were detected with CBA after 24 h of co-culture. d. Percentage of IFN-γ^+^CD4^+^ and IFN-γ^+^CD8^+^ cells after MNC-168 co-culture with PBMCs for 48 h was analyzed by Flow cytometry. e. Schematic of the experimental design: Mice were inoculated with MC38 murine colon cancer cells and gavaged with MNC-168, and intraperitoneally injected with or without anti-PD-1 after the tumor volume reached 50–100 mm^3^. f. Tumor growth curves showing changes in tumor volume after administration of MNC-168, with or without anti-PD-1. Mice per group *n* ≥11. g. Tumor growth inhibition rate was analyzed on Day 21 after inoculation of tumor cells in each group comparing with the control. h. Tumor growth curve of administration of live or Heat-Kill dead MNC-168, with or without anti-PD-1. i. Tumor growth inhibition rate analysis on Day 22. j. Schematic design for evaluating antitumor effect of MNC-168 with or without anti-PD-1 after depleted gut bacteria with ATB. k. Tumor growth curves with indicated administration of MNC-168 with or without anti-PD-1 after 2 weeks of ATB treatment. Mice per group *n* ≥ 7. l. Tumor growth inhibition rate analysis on Day 21. For all graphs, data are shown as the mean ± s.e.m.; *P < 0.05, **P < 0.005, ***P < 0.0005, ****P < 0.0001, NS, not significant. *P* values were calculated using one-way *ANOVA* with Tukey’s test for multiple comparison.

To determine whether MNC-168 modulates the response to cancer immunotherapy, we generated murine models of syngeneic tumors with MNC-168 or in combination with anti-PD-1 therapy (Figures 1(e–g)). MC38 colorectal cancer cells were inoculated subcutaneously into the mice, and MNC-168 was gavaged every day, while anti-PD-1 was intraperitoneally injected when the tumor volume reached 50–100 mm^3^ (Figure 1(e)). MNC-168 significantly reduced MC38 tumor volume and increased the tumor growth inhibition rate (TGI) (Figures 1(f–g)). Furthermore, combining MNC-168 and anti-PD-1 administration dramatically enhanced the anti-tumor effect compared to monotherapy with MNC-168 or anti-PD-1 treatment alone (Figures 1(f–g)). The observed antitumor effects of a single agent, such as an individual bacterium or Anti-PD-1 therapy, either administered alone or in combination, are consistent with the findings reported in prior studies.^14,35^ However, the tumor inhibition effect of the combination with anti-PD-1 treatment was eliminated when the heat dead MNC-168 was administrated (Figures 1(h–i)). To further determine the sole anti-tumor role of MNC-168, we depleted gut microbes in a murine MC38 tumor model using an antibiotic cocktail (ATB) 2 weeks before MNC-168 was gavaged (Figures 1(j–l)). ATB administration diminished the therapeutic effect of anti-PD-1, while MNC-168 supplementation significantly inhibited tumor growth and dramatically suppressed tumors with the anti-PD-1 combination (Figures 1(k–l)). To investigate the relationship between MNC-168 and anti-tumor activity, we gavaged the mice with dose increase of MNC-168 or combined with anti-PD-1 (Fig S3a-3b). As a result, an observable anti-tumor effect was achieved when MNC-168 was administered orally at a dose of 2 × 10^9^ CFU, but not at 2 × 10^8^ CFU. However, further increasing the dose to 2 × 10^10^ CFU did not enhance the anti-tumor efficacy. Together, these results show that MNC-168 dramatically inhibited tumor growth and improved anti-PD-1 therapy.

## MNC-168 elicits systemic immune response and enhances T cell anti-tumor immunity

**Figure 2.**
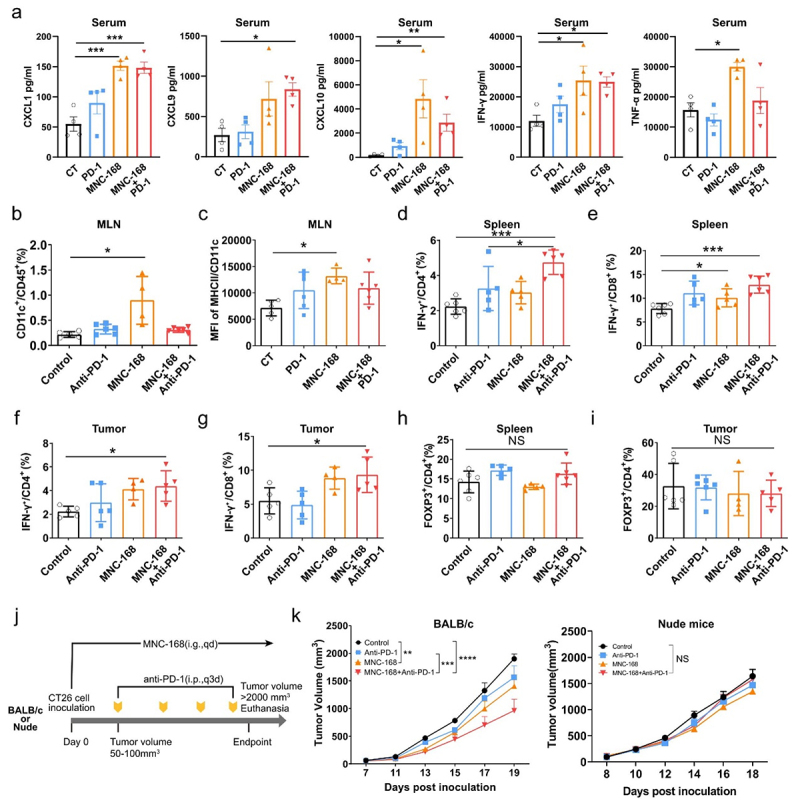
MNC-168 activates systemic immune response and enhances T cell anti-tumor immunity *in vivo*. a.

Next, we explored the mode of action of MNC-168 on anti-tumor immunity *in vivo*. First, we detected the expression of chemokines and cytokines in plasma from MNC-168 treated MC38 murine study (Figure 1(e)) using the CBA assay. MNC-168 significantly increased the levels of CXCL1, CXCL9, CXCL10, IFN-γ, and TNF-α (Figure 2(a)) *in vivo*, which was consistent with the results of immune activation of MNC-168 when co-cultured with PBMCs *in vitro* (Figure 1(c)). These results indicate a potential role of MNC-168 in systemic immune activation. Therefore, we further evaluated the effects of MNC-168 on peripheral immune organs. In the MC38 model, MNC-168 oral administration increased the number of CD11c^+^ DCs and MHCII expression of CD11c^+^ DCs in mesenteric lymph nodes (MLN) (Figure 2(b,c)), indicating the innate immunity activation role of MNC-168. In addition, the proportion of IFN-γ^+^ CD4^+^ (Figure 2(d)) and IFN-γ^+^ CD8^+^ T cells (Figure 2(e)) in the spleen was significantly increased after MNC-168 treatment. Furthermore, MNC-168 combination of anti-PD-1 further promoted the proportion of IFN-γ^+^ CD4^+^ and IFN-γ^+^ CD8^+^ T cells in the spleen compared with anti-PD-1 alone (Figure 2(d,e)). In the TME, MNC-168 significantly promoted intratumoral infiltration of IFN-γ^+^ CD4^+^ and IFN-γ^+^ CD8^+^ T cells (Figure 2(f,g)). The proportion of FOXP3^+^/CD4^+^ cells did not change in spleen and tumor tissue after MNC-168 treatment (Figure 2(h,i)).

Indicated chemokines and cytokines of plasma from different treated mice were detected by CBA assay. Each group *n* = 4. b-c. Flow cytometry analyzes the population of CD11c^+^ DCs (b) and the expression of MHCII of CD11c^+^ DCs (c) in MLN. Each group *n* ≥ 4. d-g. Flow cytometry analyzes the percentage of IFN-γ^+^CD4^+^ cells and IFN-γ^+^CD8^+^ cells derived from spleen (d-e) and tumor (f-g). Each group *n* ≥ 4. h-i. Flow cytometery analyzes the proportion of FOXP3^+^ of CD4^+^ cells in spleen (h) and tumor (i). Each group *n* ≥ 4. j. Schematic design for compering anti-tumor effect of MNC-168 between BALB/c and Nude CT26 xenograft models. k. Tumor growth curve of CT26 with indicated treatment in BALB/c (left) and Nude mice (right), respectively. Each group *n* ≥ 5. For all graphs, data are shown as the mean ± s.e.m.; *P < 0.05, **P < 0.005, ***P < 0.0005, ****P < 0.0001, NS, not significant. *P* values were calculated using two-way ANOVA(k) or one-way ANOVA with Tukey’s test for multiple comparisons (a-i).

To determine whether MNC-168 inhibits tumor growth by activating T cell-mediated anti-tumor immunity, we compared the effect of MNC-168 alone or in combination with anti-PD-1 in immune-competent and T cell-deficient mice (Figure 2(j)). Similar to the therapeutic effect previously reported, MNC-168 significantly enhanced the anti-PD-1 anti-tumor effect in the BALB/c CT26 colon tumor model, while this effect was dramatically eliminated in nude mice (Figure 2(k)). Notably, there are not any observable adverse effects on nude mice growth found at this dose of MNC-168 (Fig S4), suggesting MNC-168 administration may not induce overt systemic toxicity even in T-cell compromised hosts. Together, T-cell activation plays a crucial role in the ability of MNC-168 to mediate anti-tumor immunity in the host.

## Microbiome and metabolome analysis the influence of MNC-168 in syngeneic tumor model

To systematically investigate the impact of MNC-168 in a syngeneic tumor model, we initially used 16S rDNA sequencing to analyze the gut microbiota of subjects treated with MNC-168 and anti-PD-1 therapy (Fig. S5). We compared the diversity of gut microbiota and found that both the number of observed species and α-diversity (Shannon index) were significantly reduced following tumor cell inoculation. However, treatment with anti-PD-1 or MNC-168, either alone or in combination, restored the microbial species richness and diversity similarly to the pretumor inoculation (Fig S5a). This result is consistent with the current findings that a higher diversity of the gut microbiome is associated with improved clinical responses to ICIs immunotherapy in various cancers.^36^ PCA also showed that the composition of gut microbiota was similar between pre- and post- MNC-168 treatment groups but differed in the post-CT group (Fig. S5b). Furthermore, the linear discriminant analysis Effect Size (LEfSe) revealed a significant increase in the abundance of the *Enterococcus* genus after MNC-168 treatment (Fig. S5c). Additionally, metabolomic profiling of the cecal content revealed an increase in several molecules related to modulating immune activation following treatment with MNC-168, including inosine.^37^ and NAD^+38^ (Fig. S6a-b and Supplementary file 4). These findings suggest that MNC-168 has the potential to recover the gut microbiota and regulate immune activation, contributing to antitumor immunity.

## MNC-168 induces type I IFN response via STING pathway to promote anti-tumor immunity

**Figure 3.**
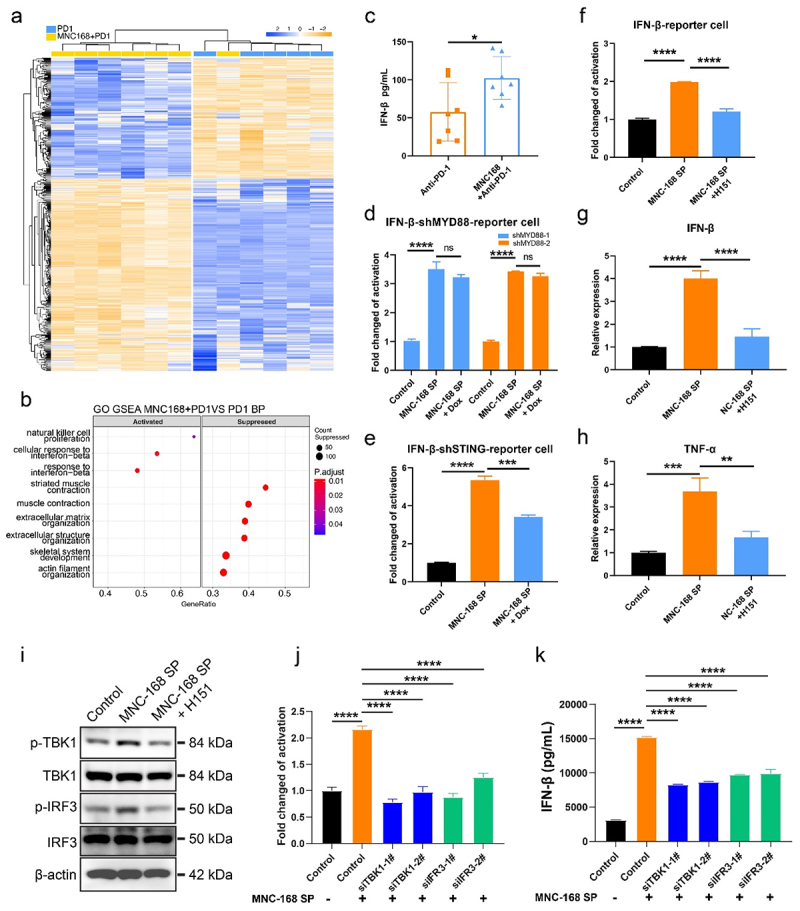
MNC-168 induces type I IFN response via STING pathway to promote anti-tumor immunity. a.

To further elucidate the potential mechanism of action of MNC-168, we performed a comparative analysis of tumor tissue transcriptomes between MNC-168 plus anti-PD-1 and anti-PD-1 monotherapy (Figure 3(a)). A total of 474 differentially expressed genes were identified, with 183 genes showing a significant increase and 291 genes exhibiting a decrease (Supplementary file 5). Furthermore, through genes enrichment analysis (GSEA), we identified that the signal pathways were not only enriched in NK cell proliferation but also focused on the interferon beta response (Figure 3(b), Supplementary file 6). To validate the role of IFN-β signaling in this process, we assessed the serum levels of IFN-β in this mouse model. The concentration of IFN-β in the serum significantly increased upon MNC-168 combined with anti-PD-1 treatment compared to anti-PD-1 treatment alone (Figure 3(c)). These findings suggest that the IFNs response may play a regulatory role in MNC-168-mediated anti-tumor immunity.

Heatmap showing differentially expressed genes between mice treated with MNC-168 combined with anti-PD-1 and anti-PD-1alone determined by RNA sequencing of tumor tissues. b. GSEA of differentially expressed genes. c. IFN-β detection of serum from anti-PD-1 and anti-PD-1 combined MNC-168 group by ELISA. d. Evaluation of IFN-β activity with THP- shMYD88-IFN-β-reporter cell through MNC-168 supernatant (10% volume) stimulation under Dox-induced knockdown of MYD88. MYD88 of THP-1-IFN-β reporter cells was induced knockdown with Dox (2 µg/mL) one day before MNC-168 supernatant treatment. Luciferase activity of the cells was detected after 24 h treatment. e and f. Evaluation of IFN-β activity was performed using THP-shSTING-IFN-β-reporter cells treated with MNC-168 supernatant under Dox-induced STING knockdown (f), in the presence or absence of the STING inhibitor H-151 (g). g and h. The mRNA expression levels of IFN-β (g) and TNF-α (h) were assessed in STING reporter cells using RT-PCR following the indicated treatments. i. Western botting of the phosphorylation of TBK1 and IRF3 in THP-1-IFN-β-reporter cells after 24 h of MNC-168 supernatant treatment with or without H-151. j. IFN-β activity evaluation of THP-IFN-β-reporter cells with TBK1 or IRF3 knockdown upon MNC-168 supernatant treatment. The TBK1 or IRF3 of cells was knocked down with two different RNA interference fragments for 24 h, respectively, and then treated with MNC-168 supernatant for another 24 h before detection. k. The IFN-β of the cells culture supernatant with indicated treatment was detected by ELISA. For all graphs, data are shown as the mean ± s.e.m.; *P < 0.05, **P < 0.005, ***P < 0.0005, ****P < 0.0001, NS, not significant. *P* values were calculated using unpaired T test for two sample comparison while one-way ANOVA with Tukey’s test for multiple comparison.

Type I interferons (IFNs) activate intracellular antimicrobial programs and influence the development of innate and adaptive immune responses.^39^ Antigen-presenting cells (APCs), such as macrophages and dendritic cells (DCs), produce type I IFNs upon sensing pathogen components and tumor antigen to primer further immune activation for prevention of infection and tumor growth.^40^ To determine the direct impact of MNC-168 on innate or adaptive immunity, we initially co-cultured T cells differentiated from human PBMCs with the MNC-168 culture supernatant. However, we found that neither 5% nor 10% MNC-168 supernatant could induce CD 4 and CD 8 T cells activation (Fig S7). Therefore, we hypothesized that MNC-168 may induce type I IFN production by APCs to promote antitumor immunity. To verify this, we evaluated potential regulatory signaling pathways using *in vitro* THP-1 models to mimic APCs. *Enterococcus* has been reported to modulate immune activation through the NOD2 signaling pathway.^18^ Therefore, we firstly assessed whether MNC-168 influences NOD2 signaling by a NOD2-specific inhibitor (Fig S8). Results of NFκB reporter cell (Fig S8a) and specific downstream gene detection (Fig S8b-8c) have demonstrated NOD2 signaling was not involved in MNC-168 activation. In addition, MYD88 is an innate immune signaling adapter, plays a vital role in the induction of type I IFN production.^41^ Using a Dox-inducible MYD88 knockdown THP-1-IFN-β-reporter cell line (Fig S10a), we found that MYD88 knockdown only slightly inhibited the effect of MNC-168 activation (Figure 3(d)). Notably, MNC-168 treatment does not influence viability of the cells (Fig S9). These results suggested that MNC-168 regulates immune activation through other mechanisms.

Since MNC-168 induce IFN-β is not major MYD88-dependent manner, it was conceivable that MNC-168 might trigger IFN-β induction through a cGAS/STING-dependent pathway, which plays an important role in resisting pathogen infection and anti-tumor immunity.^20^ Recent studies have shown that microbiota could reprogram the tumor microenvironment and enhance the ICB efficacy via STING-type I interferon-dependent.^21,22^ To test the biological activity of MNC-168 and its potential to induce downstream signaling of cGAS/STING pathway, we also generated a Dox inducible STING knockdown THP-1-IFN-β reporter cells (Fig S10b). MNC-168 supernatant significantly enhances IFN-β reporter activity, whereas STING knockdown markedly reduces IFN-β activity (Figure 3(e)). Furthermore, we assessed this role with STING specific inhibitor H-151 on MNC-168- induced IFN-β activity. H-151 significantly attenuated the IFN-β reporter activity on MNC-168 supernatant treatment (Figure 3(f)). Similarly, the mRNA level of STING targeting gene IFN-β and TNF-α was significantly up-regulated by MNC-168 supernatant, and this up-regulation was also attenuated by H-151 back to control levels (Figure 3(g,h)). To validate the cGAS/STING signaling is responsible for the IFN-β induction upon MNC-168 treatment, we detected the phosphorylation of TBK1 and IRF3, which are critical downstream effectors of STING signaling. The phosphorylation levels of TBK1 and IRF3 were increased in THP-1 cells upon MNC-168 treatment, whereas H-151 treatment prevents this signal activation (Figure 3(i)). To further confirm this signal transduction for IFN-β production induced by MNC-168, we knocked down TBK1 or IRF3 in THP-1-IFN-β reporter cells using the respective siRNA (Fig S10c). We then treated these cells with MNC-168 supernatant and detected the IFN-β reporter activity and IFN-β expression by ELISA. On MNC-168 supernatant treatment, either TBK1 knockdown or IRF3 knockdown significantly decreased not only the IFN-β reporter activity but also IFN-β protein level of the cells (Figure 3(j,k)). These findings suggested that MNC-168 induces IFN-β production through cGAS/STING/TBK1/IRF3 pathway to enhance the anti-tumor immunity.

## MNC-168 triggers STING-dependent anti-tumor immunity via secreted MVs

**Figure 4.**
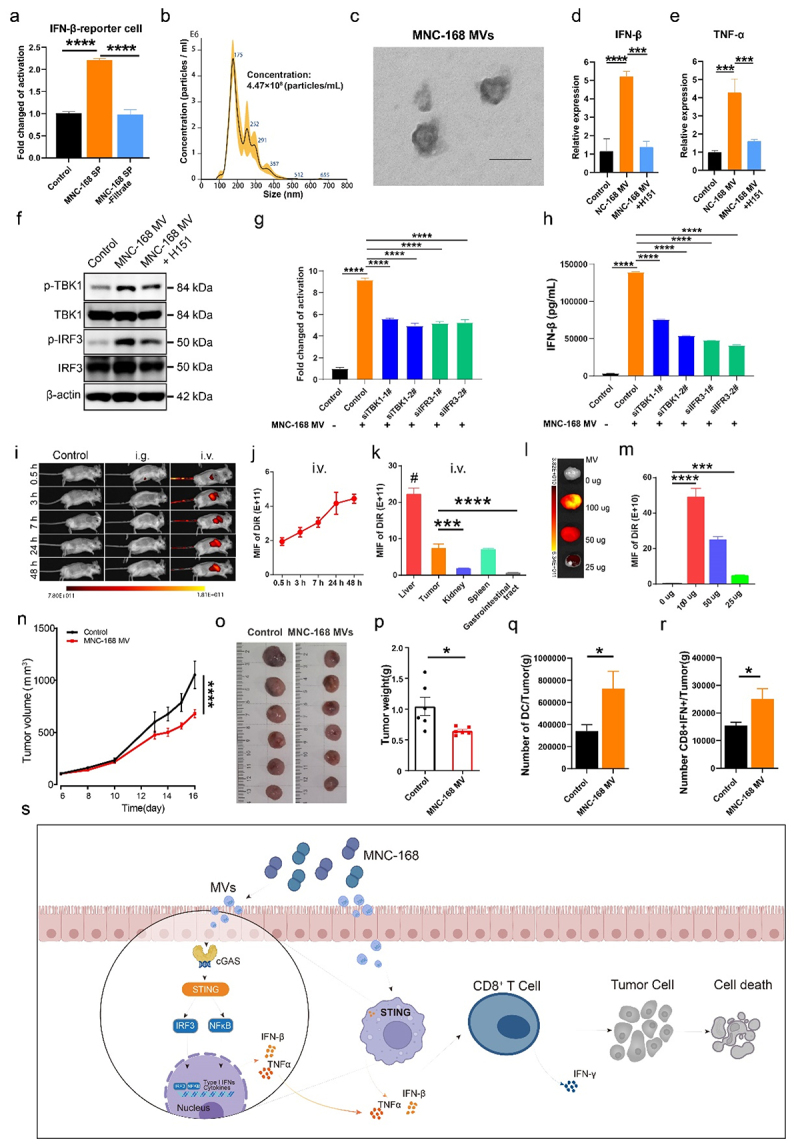
MNC-168 triggers STING dependent anti-tumor immunity via secreted MVs.

Bacteria can activate the cGAS-STING-IFN-I signaling pathway through bacterial cyclic dinucleotides (CDNs) such as c-di-AMP/GMP.^42^or bacterial membrane vesicles (MVs) that deliver bacterial DNA into host cells.^43^ To determine the active components of MNC-168, we separated the soluble small molecules (containing CDNs, LPS) and large fractions (containing large proteins and MVs) from the culture supernatant using a 100 kDa ultrafiltration device. Using the THP-1-IFN-β reporter cell assay, we found that the filtered supernatant fraction failed to activate the reporter cells compared to the unfiltered supernatant (Figure 4(a)), indicating that the active component is not soluble to small molecules. We hypothesized that the MVs derived from MNC-168 might play a significant role.

a. Comparison the activation role of MNC-168 supernatant and its filtrate which filtered by 100 KDa ultrafiltration device in THP-IFN-β-reporter cells. b. The size analysis, distribution, and particles concentration of MVs derived from MNC-168 were conducted using Nanosight. c. Morphology observation of MNC-168 derived MVs employing TEM, the scale bar represents 100 nm. d and e. The expressions of IFN-β (d) and TNF-α (e) were measured in THP-1-IFN-β-reporter cells using RT-PCR following the indicated treatments, 1 × 10.^6^ cells were seeded in 12 well plate, 2.5 × 10.^7^ particles of MVs were loaded in the presence or absence of H-151 (10 µM). f. Western botting of the phosphorylation of TBK1 and IRF3 in THP-1-IFN-β-reporter cells after 24 h of MNC-168 MVs treatment with or without H-151. g. IFN-β activity evaluation of THP-IFN-β-reporter cells with TBK1 or IRF3 knockdown upon MNC-168 MVs treatment. The TBK1 or IRF3 of cells was knocked down with two different RNA interference fragments for 24 h, respectively, and then treated with MNC-168 MVs for another 24 h before detection. h. The IFN-β of the cells culture supernatant with indicated treatment were detected by ELISA. i. Images of MNC-168 MVs in vivo tracking in CT-26 tumor model. 200 µg DiR-labeled MNC-168 MVs were administered to CT-26 tumor-bearing mice via gavage or intravenous injection. Animals were subjected to live imaging photography at indicated time-points. j. MIF of DiR analysis was performed on tumor tissue at specified time-points following intravenous injection. k. Quantitative analysis of DiR distribution in the specified organs at the endpoint dissection. l and m. Image and quantitative of DiR of tumors following a reduced-dose injection of MVs, with doses ranging from 100 µg to 25 µg at the endpoint. n. Tumor growth curves showing changes in CT26-tumor model after intratumor injection of MNC-168 MVs (10 ug/dose). *n* = 6. o and p. Image and weight quantitation of tumors. q and r. Flow cytometery analyses number of DC cells (m) and IFN-γ^+^CD8^+^ cells (n) in tumor. *n* ≥ 4. s. The mechanism model diagram of MNC-168 triggers and enhances the antitumor immunity. For all graphs, data are shown as the mean ± s.e.m.; *P < 0.05, **P < 0.005, ***P < 0.0005, ****P < 0.0001, NS, not significant. *P* values were calculated using two-way ANOVA or one-way ANOVA with Tukey’s test for multiple comparison.

To test this, we performed DNA sequencing to analyze the abundance of MNC-168 MVs in fecal samples treated with MNC-168. We observed a substantial increase in the abundance of *Enterococcus*-derived MVs following MNC-168 treatment compared to the control group (Fig S 11a). Furthermore, compared to other *Enterococcus* species, there was a notable increase in the abundance of MNC-168 MVs (Fig S11b), suggesting that MNC-168 might activate STING through secreted MVs.

To investigate further, we isolated MVs from the culture supernatant of MNC-168 and characterized their properties. NanoSight technology estimated the size of MVs to be approximately 200 nm (Figure 4(b)), although transmission electron microscopy (TEM) revealed a size range from 50 to 100 nm (Figure 4(c)), which is consistent with previous reports that NanoSight technology may overestimate particle size.^44^ Agarose gel electrophoresis demonstrated that MNC-168-derived MVs contain a significant amount of DNA (Fig S11c). Similar to the culture supernatant, MNC-168 derived MVs induced STING-dependent activation of IFN-β, as confirmed by Dox-induced STING knockdown and treatment with a specific inhibitor (Fig S11d-e). Additionally, the mRNA levels of IFN-β and TNF-α were increased in response to MVs stimulation (Figures 4(d–e)). To validate the STING signaling transduction, we also detected the phosphorylation level of TBK1 and IRF3 with MNC-168 MVs treatment. The protein level of pTBK1 and pIRF3 were increased by MNC-168 MVs treatment, whereas H-151 prevented this activation (Figure 4(f)). Moreover, knocked down TBK1 or IRF3 with siRNA, significantly prevented the IFN-β transcriptional activity and production induced by MNC-168 MVs (Figures 4(g–h)). These results are consistent with the role of the MNC-168 culture supernatant, indicating that MNC-168-secreted MVs induce IFN-β production via the STING signaling pathway.

Bacterial extracellular vesicles are capable of reaching target tissue or organs via the bloodstream, modulating specific physiological functions.^45^ Therefore, we investigate whether MNC-168 released MVs can modulate anti-tumor immunity through blood circulation. To monitor their *in vivo* distribution patterns, we employed an established approach.^46,47^ to study the trafficking of MNC-168 MVs in a CT-26 tumor model. Two hundred micrograms of DiR-labeled MNC-168 (MVs) were administered to CT-26 tumor-bearing mice via gavage or intravenous injection, which, respectively, simulated the administration route of MNC168 or the bloodborne delivery modality of MV. Regarding gavage of MNC-168 MVs, the fluorescence was detected in the stomach at 0.5 h post-administration, whereas diminished 3 h after administration, which indicates possible digestion of MVs by gastric juice (Figure 4(i), and Fig S12a). In contrast, the fluorescence of MVs can be maintained in the body for an extended period via intravenous injection. Half an hour post injection, MVs fluorescence appeared in the liver and peaked at about 3 h. (Figure 4(i), and Fig. S12a). A weak signal of MV was detected at 0.5 h. As time progressed, the fluorescence of MV within the tumor gradually intensified and reached a plateau phase after 24 h (Figures 4(i,j)). The endpoint anatomical results revealed that, while a significant proportion of MVs accumulated in the liver, there was also notable enrichment of MVs in subcutaneous tumor and spleen, compared to kidney and gastrointestinal tract (Fig12Sb and Figure 4(k)). This phenomenon is similar to current findings of bacterial MVs accumulate to a higher degree in tumor tissues, compared to other organs.^48^ Moreover, we further investigated tumor targeting using a reduced dose of MVs injection, ranging from 100 µg to 25 µg. We observed that the fluorescence intensity in the tumor decreased in a dose-dependent manner; however, a faint but detectable MVs signal remained present even at the lowest dose (Fig S12c and Figures 4(l–m)). This observation strongly supports the ability of MNC-168 MVs to extravasate and preferentially accumulate in tumor via systemic circulation to trigger the STING pathway to contribute to antitumor immunity.

As proof of concept, the role of MNC-168 in enhancing anti-tumor immunity was validated through intratumoral injection of MNC-168 MVs in a CT-26 tumor model. The administration of MNC-168 MVs resulted in a significant suppression of both tumor growth and tumor weight (Figure 4(n,p)). Furthermore, analysis of immune cells in the tumor revealed a significant increase in the number of DCs (Figure 4(q)) and IFN-γ+CD8 T cells (Figure 4(r)) in response to MNC-168 MVs treatment. These findings suggest that MNC-168 activates innate immunity and enhances T cell anti-tumor capacity. Together, these findings suggested that MNC-168 activates the STING-IFN-I pathway via bacterial MVs delivering DNA, thereby enhancing the efficacy of anti-tumor immune responses (Figure 4(s)).

## MNC-168 enhances anti-PD-1 efficacy in various preclinical tumor models and clinical ICB treatment

**Figure 5.**
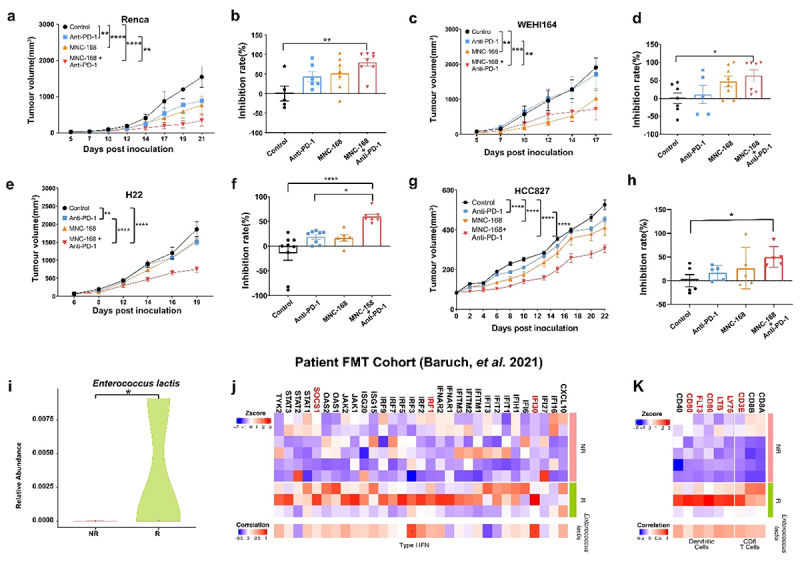
MNC-168 enhances anti-PD-1 efficacy in various preclinical tumor models and clinical ICB treatment.

Although immune checkpoint blockade (ICB) therapy has achieved favorable effects in patients, the response to ICB therapy remains low in many cancers. As live bacterial therapeutics primarily exert antitumor effects via immune activation, we employed varying syngeneic models with distinct immunogenic and genetic profiles and TME features to assess the breadth of MNC-168’s activity across heterogeneous tumor types. In the renal cell carcinoma model, a model verified with highly immune infiltration,^49^ administration of MNC-168 significantly inhibited Renca tumor growth and further improved the efficacy of anti-PD-1 treatment (Figure 5(a,b) and Fig S13a). In the fibrosarcoma model, where anti-PD-1 treatment showed minimal response in most mice, oral administration of MNC-168 significantly reduced tumor volume and dramatically enhanced the response to anti-PD-1 therapy (Figure 5(c,d) and Fig S13b). Similarly, in the H22 murine hepatocellular carcinoma model, which is characterized by an immunosuppressive tumor microenvironment,^50,51^ both single treatments with MNC-168 or anti-PD-1 had limited impact on H22 tumor growth; however, their combination led to significant suppression of tumor growth (Figure 5(e,f) and Fig S13c). Notably, MNC-168 exhibited efficacy across multiple syngeneic models (varying in immunogenicity and TME features), suggesting broad but context-dependent activity. Additionally, co-administration of MNC-168 and anti-PD-1 led to increased infiltration of CD4^+^ and CD8^+^ T cells, as well as IFN-γ^+^ positive cells, within the H22 tumor microenvironment (Fig S14a-14d), consistent with the immune activation observed in the MC38 model (Figure 2). To further evaluate the potential translatability, a humanized lung cancer model was employed to evaluate whether MNC-168 retains efficacy in a human immune context. While the humanized systems have inherent limitations (e.g., incomplete immune reconstitution and absence of human microbiota), our data suggest that MNC-168 prevented tumor growth and enhanced the anti-PD-1 therapeutic efficacy (Figure 5(g,h)). These findings suggest a potential role for MNC-168 in enhancing the efficacy of anti-PD-1 therapy across various cancer types.

a-b. Renca murine renal cell carcinoma cell syngeneic mouse model and tumor growth inhibition (TGI) rate analysis. c-d. WEHI164 murine fibrosarcoma cell syngeneic mouse model and TGI analysis. e-f. H22 murine hepatocellular carcinoma cell syngeneic mouse model and TGI analysis. g-h. CD34^+^ HSC immune system humanized mouse model of HCC827 human lung cancer cell and TGI analysis. NPSG mice, a severe combined immunodeficient mice were reconstructed the immune system with CD34^+^ HSC, HCC827 cells were inoculated, and mice were administrated with MNC-168 alone or combined with anti-PD-1. Each group of mice *n* = 5. i. *Enterococcus lactis* abundance comparison between R and NR group after FMT. j. Genes expression analysis of Type I IFN signature and correlation with *Enterococcus lactis*. k. Genes expression analysis of DCs and CD8 T cell signatures and correlation with *Enterococcus lactis*. For all graphs, data are shown as the mean ± s.e.m.; *P < 0.05, **P < 0.005, ***P < 0.0005, ****P < 0.0001, NS, not significant. *P* values were calculated using two-way *ANOVA* (a, c, e, g) or one-way *ANOVA* with Tukey’s test for multiple comparisons (b, d, f).

To further assess the clinical potential of MNC-168, we analyzed metagenomic and tumor RNA-seq data from a phase I clinical trial involving patients with anti-PD-1-refractory metastatic melanoma who underwent fecal microbiota transplantation (FMT) from complete responders, followed by re-induction of anti-PD-1 therapy.^52^ Although this clinical study is limited in scale, the efficacy and immunomodulatory outcomes are highly promising. Therefore, we compared the abundance of *Enterococcus lactis* (the Species corresponding to MMC-168) in the responder (R) group with that in the non-responder (NR) group, and found a significant increase in *Enterococcus lactis* abundance in the R group (Figure 5(i)). Moreover, we delved into the mechanism identified in our preclinical studies by comparing the transcriptome profiles from tumor between the R and NR groups and analyzing the relationship with *Enterococcus lactis* abundance. Our findings revealed that the majority of genes associated with the Type I interferon (IFN) signature were upregulated in the R group and exhibited a positive correlation with *Enterococcus lactis* abundance (Figure 5(j)). Similarly, the genes associated with dendritic cell (DC) and CD8^+^ T cell activity exhibited higher expression levels in the R group, which were positively correlated with the abundance of *Enterococcus lactis* (Figure 5(k)).

In addition, the safety of LBPs represents a critical challenge in determining their potential use as adjuvants in immunotherapy. Therefore, we conducted the toxicity study of MNC-168 on Sprague Dawley (SD) rats with gaveged at low, mediate, and high doses of MNC-168 fermental powder, respectively, to observe the possible adverse effects upon a 28-day dosed period and delayed toxicity following a 28-day recovery period. All animals survived on the scheduled day of necropsy and there were no unplanned dead or moribund animals in this study. Compared to the negative control group (0 CFU), no significant differences in body weight or food consumption were noted in male and female animals across all test groups during both the dosing and recovery periods (Fig S15). Summary data for clinical pathology analyses are presented in Supplementary file 7. Consequently, the administration of MNC-168 did not produce any observable adverse effects on the host, indicating its potential safety for future IND-enabling studies.

These data demonstrate the potential of *Enterococcus lactis* to augment the therapeutic efficacy of clinical anti-PD-1 therapy, reinforcing our preclinical observations that MNC-168 activates anti-tumor immunity through STING signaling to enhance the effectiveness of anti-PD-1 therapy.

## Discussion

Indeed, despite significant advancements in cancer therapy in recent years,^53^ a substantial proportion of patients still do not respond favorably to current treatment options.^9^ Therefore, it remains an urgent and unmet clinical need to treat those cancer patients who are resistant to current therapies. Emerging evidence has highlighted the pivotal role of gut microbiota in modulating the response to cancer treatments, including immune checkpoint inhibitors (ICIs).^54,55^ In this study, we found *Enterococcus spp* are abundant in patients who respond favorably to ICIs. Specifically, we identified MNC-168, an *Enterococcus lactis* strain, as a promising candidate that enhances the efficacy of anti-PD-1 treatment by eliciting a robust systemic immune response and augmenting T cell-mediated antitumor immunity. Mechanically, we elucidated a novel direct pathway whereby MNC-168 activities the STING-IFN-I pathway through the secretion of bacterial membrane vesicles (MVs) to target the tumor and enhance antitumor immunity, which distinguished from previous reports on bacterial CDNs in STING activation,^21^ flagellin of *Enterococcus gallinarum* MRx0518 in TLR5 activation.^56^ and SagA of *Enterococcus* in NOD2 activation.^18^ Furthermore, we validated the augmenting effect of MNC-168 on anti-PD-1 therapy across multiple preclinical cancer models, reinforcing its translational potential. Importantly, our analysis of published clinical data also suggests that *Enterococcus lactis* may augment the response to anti-PD-1 therapy in patients, further underscoring the clinical relevance of our findings. Collectively, these results position MNC-168 as a novel live biotherapeutic product with the potential to improve the efficacy of immunotherapy for cancer. By harnessing the immunomodulatory properties of gut microbiota, MNC-168 offers a promising avenue for developing more effective and personalized cancer treatments.

Microbiota is widely recognized to play an important role in cancer, influencing tumor initiation and development in either a positive or negative way, depending on the situation, species, and strains.^57^ Immunotherapies, particularly immune checkpoint inhibitors (ICIs), have dramatically improved the survival rates of patients with metastatic malignancies.^58^ Blockade of immune checkpoint molecules, such as PD-1/PD-L1 and CTLA-4, unleashes the antitumor immunity of T cells to combat diverse metastatic tumors.^59,60^ Although ICIs therapies have achieved great effects on cancer treatment, only a minority of patients (20–40%) have responded positively to PD-1/PD-L1 blockade therapy.^9,61^ Recent evidences have linked the microbiota to immunotherapy, which can improve host immunity, both locally and systemically. Specific bacterial species and strains, such as *Bifidobacterium*,^13^ *A. muciniphila*^16^ and *Faecalibacterium*.^26^ play an immune adjuvant role in anti-PD-1 immunotherapy by inducing innate and adaptive immune responses. Using high-throughput sequencing approaches, distinct microbial species or strains have been identified that positively correlate with antitumor therapy, combating cancer in the gut or at sites distant from the gut by strengthening the antitumor immunity of the host.^62,63^ When comparing the composition of gut microbiota between responders and non-responders to immunotherapy, we particularly found that *Enterococcus spp*. were enriched in responders. Notably, while certain strains of *Enterococcus spp*. have been utilized as probiotics.^64^ or exhibit protective effects against cancer,^18^ it has been reported that in specific contexts, *Enterococcus spp*. can become pathogenic,^32^ even contributing to the development of colorectal cancer (CRC).^65^ *Enterococcus faecalis* and *Enterococcus faecium* are the predominant pathogenic species within the *Enterococcus* genus encountered in clinical settings, responsible for a range of infections and contributing to drug resistance in patients.^33,34^ Although the safety and preliminary efficacy of some *Enterococcus spp*. have been validated in clinical studies for cancer therapy, such as *E. gallinarum* MRx0518 (Clinical trial information: NCT03934827), the concern over safety persists as a critical issue for LBPs. Therefore, we assessed potential risks by comparing the genome similarity, virulence factors, and resistance genes between our strains and pathogenic strains isolated from clinical settings. Our results demonstrated distinct genetic differences between the two groups of strains, with significantly lower levels of virulence factors and resistance genes in our strains. Furthermore, with further immune activation screening and tumor model evaluation from multiple aspects, including live and heat kill bacteria, different doses, gut microbes deletion, and anti-PD-1 combination, we identified MNC-168 one of our isolated *Enterocuccs. spp*. dramatically inhibits tumor growth and increases the efficacy of PD-1 blockade. Therefore, MNC-168 is a promising anticancer agent for combination treatment with immunotherapy.

Gastrointestinal (GI) tract is the primary habitat for microbes, where they can influence the host immunity by interplay with gut mucosa, mesenteric lymph nodes, and systemically.^54^ In this scenario, gut microbiota or their metabolites can activate local DCs, which then migrate to the lymph nodes to further stimulate naive T cells to differentiate into effector T cells, Tregs, or Th17 cells. These differentiated T cells can migrate back to the gut mucosa or enter systemic circulation, modulating the host’s immune response.^66,67^ In our study, we demonstrated that MNC-168 could elevate the systemic levels of CXCL9, CXCL10, IFN-γ and TNF-α. In addition, MNC-168 increased the number of CD11c^+^ DCs and the expression of MHCII on these DCs in mesenteric lymph nodes. This, in turn, promoted T cell activation both in the spleen and tumor tissue, enhancing the antitumor capacity. Therefore, in a nude mouse model bearing CT26 tumors, where T cell function is deficient, the antitumor effects of MNC-168 were abolished, both as a monotherapy and in combination with anti-PD-1 therapy. This suggests that the antitumor efficacy of MNC-168 is dependent on the presence of functional T cells.

Notably, mice and humans diverged between 65 and 75 million years ago, leading to extensive differences in their immune systems.^68^ Therefore, these may influence the efficacy of immune activation and safety profile of MNC-168. For example, although the ratio of T cells to enterocytes in the small intestinal epithelium is comparable between mice and humans, the profiles of T cells display notable differences between the two species, which influenced by age and environmental factors.^69^ This may lead to distinct T cell activation efficacy between the two species following MNC-168 administration. Therefore, humanized animal models, such as those featuring a humanized immune system or humanized gut microbiota, warrant further evaluation regarding the role of live bacterial therapeutics in the future.

The gut microbiome represents a dynamic ecosystem, where interactions among microbial species (e.g., constituents, products, and metabolites), host genetics, and environmental factors are likely to modulate treatment outcomes. For example, a higher diversity of gut microbiota is associated with favorable responses to immunotherapy in clinics. In advanced NSCLC, patients with a higher diversity of gut microbiome were more sensitive to anti-PD-1 therapy and exhibit enhanced memory T cell and natural killer cell signatures in the periphery.^70^ Similar results have been observed in immunotherapy studies of both HCC^71^ and RCC.^72^ In our study, we found that MNC-168 treatment could restore the gut microbial species richness and diversity, which is consistent with the impact of gut microbiome in improving the ICIs efficacy.

In addition, bacterial metabolites, short-chain fatty acids (SCFAs) enhance immunity by activating plasma cells to secrete IgA.^73^ or modulating CD8^+^ T cell responses to improve adoptive immunotherapy for cancer.^74^ In addition, pathogen-associated molecular patterns (PAMPs), like LPS and flagellin, derived from microbes, interact with host pattern recognition receptors (PRRs), including toll-like receptors (TLRs), NOD-like receptors (NLRs), RIG-I-like receptors, and STING, to activate the innate immune response, which further induces the activation of the adoptive immunity to resist pathogens or cancer.^40,75^ In our study, we observed a significant increase in certain metabolites, such as inosine and NAD^+^, in cecal contents when treated with MNC-168. These metabolites regulate T cell activation and enhance the antitumor efficacy of ICB.^37,38^ Further investigation into the molecular mechanisms revealed that the regulation of MNC-168 may not solely rely on NOD2 signaling. Furthermore, transcriptome analysis revealed a significant enrichment of the response to interferon-beta, an item in the KEGG pathway, in the activated group. Additionally, there was a significant increase in IFN-β levels in serum when MNC-168 was combined with anti-PD-1 treatment compared to anti-PD-1 treatment alone, indicating that IFN-beta signaling may be involved in this process. The cGAS-STING-type I IFN signaling pathway plays a crucial role in activating host innate and adaptive immunity against pathogen invasion and cancer initiation.^76,77^ Therefore, STING is a potential target for cancer therapy, which many natural and synthetic STING agonists were developed to augment response rates to current immunotherapy approaches and overcome acquired resistance in clinical therapy.^78^ Recently, Lam et al. discovered that bacterial CDNs induce STING-mediated type I IFN activation in intratumoral monocytes, reprogramming the tumor microenvironment to improve the efficacy of immune checkpoint blockade,^21^ which emphasized the potential role of STING signaling in immunotherapy. Moreover, an engineered *E. coli Nissle* Strain, SYNB1891.^79^, expressing STING agonist, demonstrated safety and induced type I IFN-associated pharmacodynamics in tumor and peripheral blood in a phase I trial.^80^ Therefore, we aimed to evaluate whether MNC-168 modulates STING signaling. Our study found that MNC-168 specifically activated STING signaling pathway in THP-1 cells through its culture supernatant, suggesting a potential role of bacterial components or products in this process. Further investigation revealed that the activation of STING signaling can be attributed to the MVs derived from MNC-168, rather than the small molecules present in the MNC-168 supernatant. Through in vivo tracking, we demonstrated that MNC-168 released MVs specific targeting tumor tissue, suggesting the potential antitumor mechanism. As proof of concept, we evaluated the antitumor effect of MNC-168 MVs by intratumor injection in a CT-26 tumor model. The administration of MNC-168 MVs significantly suppressed tumor growth, leading to a significant increase in the number of dendritic cells (DCs) and IFN-γ^+^CD8^+^ T cells, suggesting the immunomodulatory effects of MNC-168 MVs through STING signaling.

Although STING activation serves as a protective mechanism for the host, excessive or aberrant activation can lead to chronic inflammation or autoimmune diseases. For intrinsic factors, such as systemic lupus erythematosus (SLE), a multifactorial chronic autoimmune disease. Emerging evidence indicates that the cGAS-STING pathway plays a pivotal role in the pathogenesis of SLE. Deficiencies in TREX1, RNaseH2C, and DNase I lead to aberrant accumulation of cytosolic DNA, thereby amplifying the type I IFN signaling cascade through the cGAS – STING pathway, exacerbating inflammation and damaging multiple organs.^81^ For extrinsic factors, such as severe COVID-19 disease, the SARS-CoV-2 spike protein-induced cell fusion activates the cGAS-STING-IFN pathway, which may contribute to the excessive inflammatory response observed in the lungs of these patients.^82^ Therefore, excessive activation of STING is detrimental to the host. Unlike systemically administered STING agonists, MNC-168, derived from healthy individuals, is designed for oral administration, and its safety has been verified through toxicity studies in Sprague Dawley (SD) rats.

Sex-specific differences in gut microbiota composition represent a critical factor that warrants consideration in human diseases, including cancer.^83^ In our study, male mice were primarily utilized due to the higher prevalence of specific tumor types in males.^84^ and these models are well-established in cancer research. Moreover, the STING signaling activated by MNC-168 has not been observed to exhibit sex-biased differences. Nevertheless, given the potential impact of sex differences in gut microbiota on cancer, this bacterial-based interventions might exhibit distinct effect in two sexes. For instance, *Carnobacterium maltaromaticum*, which is specifically depleted in female patients with CRC, protects against CRC in females through an estrogen-SLC3A2-vitamin D-VDR dependent mechanism.^85^ However, this protective effect is lacking in males. Therefore, these findings highlight the significant implications of sex-related differences in gut microbiota for cancer development and treatment, necessitating careful consideration when designing LBP interventions.

Immune checkpoint blockade (ICB) therapy has already acquired a favorable effect in patients; however, the response of ICB therapy is still low in many cancers,^86^ due to the reason of distinct immunogenic and genetic profiles and TME features, which preventing the effect of immunotherapy. Therefore, we employed varying syngeneic models to assess the breadth of MNC-168’s activity across heterogeneous tumor types, such as renal cell carcinoma, fibrosarcoma, hepatocellular carcinoma. MNC-168 prevented tumor growth and enhanced the anti-PD-1 therapeutic efficacy in these varying models. Although the syngeneic murine models serve as the workhorse in cancer immunology.^87^ for candidate immunotherapeutic drugs development^88^ and mechanism study,^89^ these models often inadequately replicate human tumor immunobiology, particularly in microbiome-dependent contexts.^90^ Therefore, we try to utilize the CD34^+^ hematopoietic stem cells reconstituted humanization model to evaluate the effect of MNC-168, our data suggest that MNC-168 retains efficacy in a human immune context. Furthermore, with analysis of a phase I clinical trial data, we found that the abundance of the MNC-168 species, *Enterococcus lactis*, was higher in responder patients of anti-PD-1 and positively relate with Type-I-IFN, DCs, and CD8 T cell signature genes. These data support our preclinical findings and suggest potential applications of MNC-168 in cancer immunotherapy.

It is important to note several limitations in our study. Although we demonstrated that MNC-168 promotes antitumor immunity via MVs targeting innate immunity within the tumor microenvironment (TME), the specific cell types receiving these signals require further clarification in future investigations. Additionally, future work needs to delineate the relative contributions of myeloid versus lymphoid cells to these effects. While our focus was on the STING-type I IFN signaling pathway, the potential roles of gut microbiota and bacterial metabolites should not be overlooked. Notably, our data indicate that MNC-168 administration also modulates the microbiome and metabolome toward a more favorable antitumor profile. Therefore, comprehensive elucidation of MNC-168‘s function necessitates further investigation into these aspects. As a potential novel Live Biotherapeutic Product (LBP), MNC-168 has demonstrated antitumor efficacy across multiple preclinical tumor models. However, the paucity of paired gut microbiome and tumor RNA-seq datasets from human cancer patients hindered robust associations between *Enterococcus lactis* abundance and the IFN-I phenotype across different cancer types. Validating and extending these findings to larger, diverse patient cohorts will be crucial. Despite these limitations, our study identifies *Enterococcus lactis* MNC-168 as a novel LBP for cancer immunotherapy and advances our understanding of microbiota-mediated regulation of antitumor immunity, offering potential avenues for enhancing therapeutic efficacy.

In conclusion, we demonstrated that MNC-168 not only had antitumor capacities but also enhanced the efficacy of anti-PD-1 treatment in multiple refractory tumor models by activating both innate and adaptive immunity. Further elucidation of the molecular mechanism revealed that MNC-168 triggered activation of the STING pathway via its secreted MVs, thereby enhancing antitumor immunity. These findings emphasize that MNC-168 could be a novel live biotherapeutic product with potential for cancer therapy and the ability to improve the efficacy of current immunotherapy.

## Materials and methods

### Bacteria

*Enterococcus spp*. strains including MNC-168 were supplied by Moon (Guangzhou) Biotech Co., Ltd. which isolated from healthy human donors. *Enterococcus lactis* MNC-168 was deposited under No. GDMCC NO: 61121 in Guangdong Microbial Culture Collection Center at Guangdong Institute of Microbiology. All *Enterococcus spp*. strains were inoculated into self-optimized medium MM01 (peptone, 15 g; glucose, 20 g; yeast extract, 15 g; cysteine, 1 g; sodium acetate, 5 g; sodium citrate, 4 g; dipotassium phosphate, 2 g; magnesium sulfate, 0.1 g; manganese sulfate, 0.05 g; Tween 80, 1 g contained per liter; pH 6.3–6.5) and incubated under anaerobic conditions at 37°C for 24 h. All processing of bacteria was performed in an anaerobic platform and all the reagents were vented in an anaerobic atmosphere for at least 24 h prior to use.

## Microbiome enrichment analysis between patients with response and non-response to immunotherapy

The data for analyzing the microbiome enrichment between response and non-response to immunotherapy were originated from the clinical cohort (Routy et al., 2017: *n* = 249 patients; Matson et al., 2018: *n* = 42 melanoma patients). Key confounders were accounted in these studies including antibiotic use, detailed patient histories. Alpha diversity was calculated to estimate the species richness of a sample using Shannon index and Simpson index, which were calculated using R package ‘vegan’ (ordination methods, diversity analysis, and other functions for community and vegetation ecologists), based on species relative abundance. Beta diversity was calculated, using sample-sample distance (including the Jensen-Shannon distance and the Bray distance), to estimate the difference in species richness between different samples based on species relative abundance. Bacterial taxonomic comparisons were estimated using Wilcoxon rank sum test between R vs. NR groups based on the species relative abundance, and the significantly different species were determined by Wilcoxon rank sum test (*p* < 0.05).

## Genomic similarity, virulence factors and resistance gene analysis of *Enterococcus*. Spp. between clinical source and our isolated strain

Genomes of clinical strains were download from NCBI Pathogen Detection (genus limited to “Enterococcus,” isolation source limited to “hospital,” isolation type limited to “clinical”). The average nucleotide identity (ANI) analysis among strains in this study and clinical strains was conduct by fastANI (Version 1.33). Genomes of clinical strains were then analyzed for genetic coding by the same method used in this study. Virulence factors were identified by NCBI blastp (Version: 2.7.1+, Database: VFDB, updated on 2019/09/19). Antibiotic Resistance was identified by RGI pipeline (Version: 6.0.4).

## Cell culture

The MC38 and H22 cells were purchased from Nanjing Kebai Biotechnology. The Renca, WEHI164, CT26, 293T and THP-1 cells were purchased from the American Type Culture Collection (ATCC). PBMCs were purchased from Milestone® Biotechnologies. All cell lines were authenticated using DNA fingerprint analysis and were negative for mycoplasma contamination. MC38 and 293T cells were cultured in DMEM medium while other cells were cultured in RPMI-1640 medium supplemented with 10% fetal bovine serum (Gibco) and 100 units per mL of penicillin (Gibco) and streptomycin (Gibco). All cells were cultured at 37°C with 5% CO_2_ atmosphere.

## Mice

C57B6/J (male) and BALB/c-Nude (male) were purchased from GemPharmatech Inc. BALB/c (male) were purchased from Shanghai Model Organisms Center Inc. All the mice were at the age of 6–8 weeks and maintained in a specific pathogen-free environment. All animal experiments were carried out according to protocols approved by the Institutional Animal Care and Use Committee of Moon Biotech Co., Ltd., in compliance with the Guide for the Care and Use of Laboratory Animals (EC202008-4T，EC202008-6T，EC202102-1T). The number of mice in this study provided sufficient statistical results, including for tumor size, cytokines/chemokines, and immune profiling, and all the mice were randomly distributed into the experimental groups for this study.

## Syngeneic tumor mouse model

For MC38 tumor model, C57B6/J male mice were subcutaneously (s.c.) injected with 2 × 10.^4^ MC38 colon cancer cells and randomly divided into control group and MNC-168 group which were, respectively, intragastric (i.g.) administered with control medium or 2 × 10.^9^ CFU MNC-168 every day following tumor cells inoculation. When the tumor volume was around 50–100 mm^3^, mice of control group and MNC-168 group were intraperitoneally (i.p.) injected with or without 10 mg/kg anti-PD-1 monoclonal antibody (mAb; clone RMP1–14, BioXCell) in 0.9% sodium chloride every 3 days for 4–6 times in total. Tumor size was measured every 2 days until the endpoint. For dose assessment in anti-tumor effect, 2 × 10.^8^ CFU, 2 × 10.^9^ CFU and 2 × 10.^10^ CFU of MNC-168 were administrated in MC38 tumor model with or without anti-PD-1, respectively. Further, for evaluating the sole role of MNC-168 in anti-tumor, gut microbes were also depleted with antibiotic cocktail (ATB including 1 mg/mL ampicillin, 5 mg/mL streptomycin, 1 mg/mL colistin) by supplementing the mice with ATB containing water 2 weeks before administration of MNC-168 with or without anti-PD-1 in MC38 tumor model. For multiple tumor models, BALB/c mice were subcutaneously injected with 5 × 10.^4^ Renca, 5 × 10.^4^ WEHI164, and 5 × 10^4^ H22 cells, respectively, to establish diverse syngeneic tumor model and administrated with MNC-168 or combined with anti-PD-1 similar to MC38 tumor model. For comparison of the anti-tumor effect of MNC-168 between immunocompetent and T cell deficient mice, BALB/c and nude mice were subcutaneously injected with 1 × 10.^6^ CT26 cells, respectively. MNC-168 (2 × 10.^9^ CFU) or combined anti-PD-1 (10 mg/kg) were administrated via intragastrically and intraperitoneally injection, respectively. To evaluate the role of MNC-168 MVs, a dose of 10 μg MVs was administered via intratumor injection in the CT-26 tumor model every alternate day for a total of four administrations.

## CD34^+^ HSC immune system humanized mouse model

Humanized female mice reconstituted with CD34+ hematopoietic stem cells (HSCs) and exhibiting hCD45+ cell engraftment exceeding 25% at approximately 16 weeks post-transplantation were subcutaneously inoculated with 5 × 10.^6^ HCC827 cells. The anti-tumor efficacy of MNC-168, either as a monotherapy or in combination with anti-PD-1 antibody, was subsequently evaluated.

## Bacterial colonization analysis

Mouse feces were collected at the endpoint of the MC-38 study. Total DNA was extracted using Fecal genome DNA extraction kit (TIANGEN, DP328) according to the manufacturer’s instructions. DNA concentrations were measured by Microplate reader. Reference plasmid containing the MNC-168 gene was subjected to a 10-fold dilution gradient for the purpose of generating a standard curve. Absolute quantification of MNC-168 was performed using real-time PCR (Applied Biosystems Quantstudio3). For total bacteria analysis, primers were used as follows: Total-F- CGGYCCAGACTCCTACGGG; Total-R- TTACCGCGGCTGCTGGCAC. For MNC-168 analysis, primer sequences were as follows: 168F-AACTTGTGTCGTATCCCTTTGTCA; 168 R-TAACCCGAAAGTTTCTGATAAAGATG; Probe: TTATGCGGAAAGAAGAT-MGB-NFQ. Quantification was calculated according to the standard curve and the CT value of the sample. The content unit of MNC-168 in the final calculated fecal sample was uniformly converted to CFU/g.

## Flow cytometry analysis

Tumor, spleen, and MLN from the mice were collected at the endpoint of the MC-38 study. Dissected tumors were cut into small pieces, homogenized using tissue crusher and digested in digestion buffer (containing 1 mg/mL collagenase IV, 20 µg/mL DNase I and 10% FBS) using shaker with 200 RPM at 37°C for 20 min and then filtered through a 70 μm cell strainer. The spleens and MLNs were mashed in PBS and filtered through a 70 μm cell strainer. Single-cell suspensions of tumor, spleen, and MLN were incubated with anti-mouse CD16/CD32 (BD 553,142) to block the Fc receptor. Cells viability was stained with LIVE/DEAD Fixable Dead Cell Stain kit (Thermofisher, L3224) followed by staining with the corresponding antibodies. The following anti-mouse antibodies were used CD45-AF700 (BioLegend 103,127), CD3-APCCY7 (BioLegend 100,222), CD4-APC (BioLegend 100,516), CD8-PC5.5 (BioLegend 100,734), CD45-PC5.5 (BioLegend 103,132), CD4-PE (BioLegend 100,512), CD45-PECY7 (Thermofisher, 25–0451–82), CD11c-PC5.5 (BioLegned 117,328), MHCII-APC (BioLegned 107,614), IFN-γ-PECY7 (BioLegend 505,826), Foxp3-APC (BD 560,401). For intracellular markers, cells were fixed and permeabilized using Cytofix/Cytoperm™ Fixation/Permeabilization buffer (BD 554,714) or FOXP3/Transcription Factor Staining Buffer kit (Thermofisher, 00–5523–00) according to the manufacturer’s instructions after surface staining and incubated with the corresponding antibodies.

The stained cell analysis was performed with DxFLEX (Backman), and the data were analyzed with CytExpert software (version 2.0.0.283). The preliminary gating strategy for all analysis is as follows: (1) FSC-A versus SSC-A for debris exclusion; (2) FSC-H versus FSC-A for doublet exclusion; (3) SSC-A versus Live/Dead for dead cell exclusion; (4) SSC-A versus CD45 for positive cells (leukocytes); (5) CD11c versus CD45 for DCs, while CD11c versus MHC II from CD45^+^ cells for DC MHC II positive analysis; (6) CD3 versus CD45 for T cells; (7) CD4 versus CD8 from CD3^+^CD45^+^ cells for CD4^+^ and CD8^+^ T cells, while IFN-γversus CD4 or CD8 from CD3^+^CD4^+^ or CD3^+^CD8^+^ T cells for IFN-γ^+^CD4^+^ or IFN-γ^+^CD8^+^ analysis; (8) Foxp3 versus CD3 from CD45^+^ cells for Treg cells.

## Cytometric Bead Array (CBA)

Supernatants of THP-1 or PBMC co-cultured with MNC-168 were collected and analyzed with CBA Kit, including IL-6 (BD 558,276), TNF-α (BD 560,112), IL-1β (BD 558,279), CCL5 (BD 558,324), CXCL10 (BD 558,280) and MCP-1 (BD 558,287). For mouse, blood from the mice eye socket vein of mice were collected in EDTA anticoagulant tube and centrifuged with 500× g, 10 min for plasma. Cytokines of mouse plasma were analyzed by a LEGENDplex Custom Mouse Panel Kit, including IFN-γ, IL-2, IL-1β, CXCL10(IP10), CXCL1(KC), TNF-α, CXCL9(MIG), and IL-6 (Biolegend, US, CLPX-200511OY-GENEMA).

## Immunohistochemistry

For histologic analysis, tumor specimens of H22 mouse model were fixed with 4% paraformaldehyde, dehydrated in ethanol, embedded with paraffin, and stained with hematoxylin and eosin (H&E) and stained with CD4 (SouthernBiotech, 1540–01), CD8 (Servicebio, GB13429), IFN-γ (BioLegend 606,853) and Foxp3 (BioLegend 126,406) antibodies, respectively. Image acquisition was using Panoramic section scanner (Hungary, 3DHISTECH). Positive stained cells in an area of the slide were analyzed and calculated using Halo (v3.0.311.314) software with Indica labs – Multiplex IHC v2.2.0 module Sample preparation and image acquisition were conducted by Wuhan servicebio technology Co., Ltd. The quantification and graphical representation of data were conducted using GraphPad software (version 8.0.1).

## Transcriptomic profiling of tumor

At the endpoint of treatment model, tumors were collected and the total RNA was extracted with MiniBEST Universal RNA Extraction Kit (Takara, 9767) following by constructing the library for transcriptome sequencing. The following process of RNA-sequencing was conducted by Novogene (Beijing, China). For analysis, briefly, RNA-sequencing reads were filtered to obtain high-quality reads using fastp (version 0.20.0) software. This process includes removing sequencing adapters, primer dimers, sequences which containing N bases >5 and trimming bases with a quality score < Q15. Then, using HISAT2 (version 2.2.1) software, trimmed reads were mapped to GRCm38 (Ensembl release 101) reference genome. For the differentially expressed genes (DEGs) enrichment analysis, the featureCounts function (version 2.0.1) was used to count transcript features from all samples, and sum the counts to the gene level. Then, differential expressed genes (DEGs) between each group were calculated by the DESeq2 R package. Subsequently, Gene Ontology (GO) and Gene set enrichment analysis (GSEA) were performed using the clusterProfiler based on the gene set from org.Mm.eg.db.

## Metabolomic profiling of cecal content

Metabolomic profiling was conducted by Novogene (Beijing, China). Briefly, metabolites were extracted from cecal content with prechilled 80% methanol and 0.1% formic acid and further clean with 60% methanol LC-MS grade water following injected into the LC-MS/MS system analysis. UHPLC-MS/MS analyses were performed using a Vanquish UHPLC system (ThermoFisher, Germany) coupled with an Orbitrap Q ExactiveTM HF mass spectrometer (Thermo Fisher, Germany) in Novogene Co., Ltd. (Beijing, China). For metabolite identification, the raw data were processed using the Compound Discoverer 3.1 (CD3.1, ThermoFisher). The normalized data were used to predict the molecular formula, and the peaks were matched with the mzCloud (https://www.mzcloud.org/), mzVault and MassList database. Statistical analyses were performed using the statistical software R (R version *R*-3.4.3), Python (Python 2.7.6 version) and CentOS (CentOS release 6.6). These metabolites were annotated using the KEGG database (https://www.genome.jp/kegg/pathway.html.), HMDB database (https://hmdb.ca/metabolites.) and LIPIDMaps database (http://www.lipidmaps.org/). The univariate analysis (t-test) was used to calculate the statistical significance (P-value). The metabolites with VIP >1 and P-value < 0.05 and fold change ≥ 2 or FC ≤ 0.5 were considered as differential metabolites. The correlation between differential metabolites was analyzed by cor in R language (method = pearson).

## 16S rRNA sequencing

Fresh fecal pellets collected from individual mice were stored at −80°C until analysis. DNA extraction was performed by Fecal genome extraction kit (Tiangen, DP328), which followed by 16S ribosomal RNA (rRNA) gene amplicon sequencing at the Novogene (Beijing, China) using their standard workflow. Samples were sequenced on the Novaseq 6000 platforms.

For analysis, briefly, paired-end reads were filtered by fastp software to obtain high-quality reads and were merged using bwa pemerge function. High quality reads were further analyzed using the Quantitative Insights Into Microbial Ecology (QIIME2 version 2019.7) bioinformatics pipeline. In our study, DADA2 was utilized to generate amplicon sequence variants (ASVs) by denoising, removing chimeric and short reads. Furthermore, SILVA v138_99 16S rRNA gene database was used to train Naïve Bayesian classifier for taxonomic classification of ASVs. The biodiversity of the samples, including alpha and beta diversity, was calculated using the q2-diversity plugin. Finally, the linear discriminant analysis (LDA) effective size (LefSe) was conducted to identify the significantly different species.

## Plasmids

For reporter gene plasmid construction, the NFκB reporter gene and IFN-β reporter gene were acquired by referring the reporter plasmid 4x NFkB Luc (Addgene, Plasmid #111216) and IFN-β promoter_pGL3 (Addgene, Plasmid #102597), respectively. The reporter genes were subcloned into a lentivirus plasmid pCMV-Puro which deleted the original CMV promoter to construct the lentivirus reporter plasmid (pNFkB-Luc-puro and p IFN-β promoter-Luc-puro).

## Reporter cell engineering

Lentiviruses containing NFκB-Luc or IFN-β promoter-Luc reporter genes were produced in 293T cells through co-transfection of pNFκB-Luc-puro or pIFN-β promoter -Luc-puro with psPAX2 and pMD2.G plasmids. To generate THP-1-NFκB-reporter or THP-1-IFN-β reporter cell lines, THP-1 cells were infected with the above packaged lentiviruses and the stable clone cell line was selected by puromycin (2 µg/mL). For gene inducible knockdown cell engineering, fragments of shMYD88 or shSTING were constructed into pLKO-tet-zeocin plasmid and the lentiviruses were produced in 293T cells following the above method. Then, the THP-1-shMYD88-IFN-β reporter cell line and THP-1-shSTING-IFN-β reporter cell line were constructed by infecting the above packaged lentiviruses of pLKO-tet-shMYD88 and pLKO-tet-shSTING, respectively. Stable cell lines were selected with zeocin (300 µg/mL) about 2 weeks.

## RNA interference

THP-1-IFN-β-reporter cells were seeded in 24 wells plate, two distant siRNA fragment (50 µM) of TBK1 or IRF3 were transfected with Lipofectamine™ RNAiMAX (Thermo fisher 13,778,100). Post 24-h transfection, cells were used for RNA knockdown efficiency detection or downstream experiments. SiRNA fragments were purchased from RIBOBIO, China. Sequences of the siRNA, siTBK1–1#: CCACAAATTTGATAAGCAA; siTBK1–2#: GAAGAAATATGGAGCAACA; siIRF3–1#: GTGGACCTGCACATTTCCA; siIRF3–2#: AGACATTCTGGATGAGTTA.

## Reporter gene activity assay

THP-1-NFκB reporter or THP-1-IFN-β reporter cells were seeded in white flat 96 well plate at 1 × 10.^5^ cell/well. After 24 h treatment with MNC-168 supernatant or MVs, the fluorescence detection was performed according to the manufacturer’s instructions of Luminescent Kit (Promega, E2610) on the Microplate Reader.

## RT-qPCR analysis

Total RNA was extracted using MiniBEST Universal RNA Extraction Kit (Takara, 9767) following the instruction manual. For reverse-transcription, 2 μg total RNA was reverse-transcribed to cDNA with PrimeScript™ RT reagent Kit with gDNA Eraser (Takara, RR047A). Human GAPDH gene was used as an internal control. Real-time PCR was performed with Applied Biosystems Quantstudio3 Real-time PCR machine and PrimeScript RT Enzyme Mix reagent (Takara, RR037Q) with the following primers:

qh-IL1β-F: CCACAGACCTTCCAGGAGAATG

qh-IL1β-R: GTGCAGTTCAGTGATCGTACAGG

qh-TNFα-F: GAGGCCAAGCCCTGGTATG

qh-TNFα-R: GGGCCGATTGATCTCAGC

qh-IFNβ-F: ATGACCAACAAGTGTCTCCTCC

qh-IFNβ-R: GGAATCCAAGCAAGTTGTAGCTC

qh-GAPDH-F: ACAACTTTGGTATCGTGGAAGG

qh-GAPDH-R: GCCATCACGCCACAGTTTC

qh-STING-F: CCTGAGTCTCAGAACAACTGCC

qh-STING-R: GGTCTTCAAGCTGCCCACAGTA

qh-MYD88-F: GAGGCTGAGAAGCCTTTACAGG

qh-MYD88-R: GCAGATGAAGGCATCGAAACGC

qH-TBK1-F: CAACCTGGAAGCGGCAGAGTTA

qH-TBK1-R: ACCTGGAGATAATCTGCTGTCGA

qH-IRF3-F: TCTGCCCTCAACCGCAAAGAAG

qH-IRF3-R: TACTGCCTCCACCATTGGTGTC

## Western bolting

Total protein of the cells was extracted with RIPA lysis buffer containing protease inhibitor cocktail and phosphatase inhibitor cocktail (Yeasen, 20214ES03, 20109ES05). Protein quantification was detected by the BCA (Beyotime, P0012). Equal amounts of protein were separated by SDS-PAGE and transferred to PVDF membrane (Millipore). Membranes were blocked in 5% BSA and incubated with relevant primary antibodies at 4°C overnight. The membrane was washed with TBST and incubated with secondary HRP antibodies. Next, the membrane was then washed with TBST. ECL was applied for film development. Antibodies were used as follows: Phospho-TBK1/NAK (Ser172) (D52C2) XP® Rabbit mAb (CST, 5483), TBK1/NAK (D1B4) Rabbit mAb (CST, 3504), Phospho-IRF-3 (Ser386) (E7J8G) XP® Rabbit mAb (CST 37,829), IRF-3 (D6I4C) XP® Rabbit mAb (CST 11,904),β-actin Rabbit mAb (SDT-R015) (STARTER, S0B0005) Goat anti-Rabbit IgG(H+L), HRP (STARTER, S0B4002).

## ELISA assay

For mouse IFN-β assay, serum from the mouse tumor model was collected and the concerntraion of IFN-β was measured with Mouse Interferon β,IFN-β/IFNB ELISA Kit (CUSABIO, CSB-E04945m). For cell supernatant assay, cell culture medium was collected and measured with Human Interferon β,IFN-β/IFNB ELISA Kit (CUSABIO, CSB-E09889h).

## Bacterial membrane vesicles isolation

Bacterial MVs were isolated from MNC-168 culture medium by commercial Bacterial MVs isolation kit (Rengen Biosciences, BacMV10–10). Briefly, 10 mL of culture medium was fully mixed with 1 mL Bind buffer. Then, 400 µL Binding Resin was added into the above mixture and mix upside down at room temperature for 15 min. Centrifuge the above mixture at 1,500× g, for 2 min. Discard supernatant and transfer the resin to the purification column and wash the MVs with washing buffer for two times. Finally, MVs were eluted by Elution buffer. The concentrated bacterial MVs could be used for further experiments or stored at −80°C.

## Fecal membrane vesicles DNA extraction and metagenomic sequencing

The fecal membrane vesicles were isolated and lysed, followed by extraction of vesicle DNA through isopropyl alcohol precipitation. Subsequently, the concentration and quality of DNA were determined using Qubit 4.0 and NanoDrop spectrophotometer, respectively, while the integrity of DNA was assessed through 1% agarose electrophoresis. The library was generated by fragmenting the DNA and ligating adapters, followed by target fragment enrichment. The size and concentration of fragments were measured using Qseq400 and Qubit 4.0, respectively. Finally, Illumina NextSeq 2000 was employed for DNA sequencing.

## Bacterial membrane vesicles abundance analysis

Metagenomic data were analyzed with Kaiju and the representative genomes of bacteria, archaea, and viruses in NCBI RefSeq as references, conducted a relative abundance analysis comparing the MNC-168 treated group to the control group. Specifically, compared the relative abundance of Enterococcus between these two groups. DiTASiC was employed to analyze the level of abundance for different strains within Enterococcus.

## Nanosight measurement

MVs size was measured by the NanoSight NS300 analyzer (Malvern Instruments Ltd, Malvern, UK). Briefly, isolated MVs were diluted 200 times with PBS, and the size, distribution, and concentration of MVs was analyzed with NanoSight NS300 combined with the software NTA 3.4 Build 3.4.003.

## Transmission Electron Microscopy (TEM)

MVs morphology observation was performed by TEM using negative staining. Briefly, 5 μL of isolated MVs was deposited onto copper mesh. After incubation for 1 min, they were stained with 1% phosphotungstic acid for 2 min, rinsed with pure water for twice, air-dried at room temperature for 10 min, and subsequently examined under transmission electron microscopy (Tecnai G2 Spirit Bio TWIN) with 80 kV voltage.

## MVs DNA electrophoresis

The MVs of MNC-168 were lysed at 95°C for 5 min and subsequently treated with RNAase for 10 min, followed by detection using a 1% agarose gel for electrophoresis.

## In vivo tracking of MNC-168 MVs

The fluorescent dye 1, 1’-dioctadecyl-3, 3, 3,’ 3’-tetramethylindotricarbocyanine iodide (DiR) (Yeasen, 40757ES25) was used to label MNC-168 MVs. Purified MVs were incubated in the presence of 5 mM DiR for 20 min at 37°C, then the MVs were washed with PBS ultrafiltration for three times to remove the unbounded dye. Finally, the labeled MVs were resuspended in PBS prior to use. CT-26 tumor model was built according to the previous method, when the tumor volume reached 400–500 m^3^, the labeled MVs (200 ug) were injected intravenously or gaveged to the mouse and monitor the fluorescence in mouse at different timepoint using the IVIS spectrum (Tanon ABL X5). Post 48-h administration, the tumor-bearing mice were anesthetized with pentobarbital and the fluorescence images of the organ and tumor were captured using IVIS spectrum. For doses injection study, the labeled MVs from 100 ug to 25 ug were injected intravenously and monitor the fluorescence in mouse at different timepoint, which anesthetized post 48 h for imaging the fluorescence in tumor.

## Patient metagenome analysis

Metagenome data obtained from 3 R and 7 NR anti-PD-1-refractory metastatic melanoma patients treated with fecal matter transplant (FMT) and reinduction of nivolumab (anti-PD-1), which is a phase 1 clinical trial (NCT03353402), the primary objectives were to assess the safety and feasibility (Baruch et al., 2021). Key confounders were accounted in this study including antibiotic use, dietary control, and genetic factors. Raw reads were quality trimmed using fastp (v0.19.7) and filtered against the human genome (hg19) using Bowtie2 (v2.3.4). After quality trimming and filtering, the clean reads were used in downstream analyses. The taxonomic classification of bacteria was assigned to metagenomic reads using Kraken 2 (v.2.1.2), an improved metagenomic taxonomy classifier that utilizes k-mer-based algorithms. A custom database consisting of bacterial reference genomes from the NCBI RefSeq database (accessed in January 2023) was built using Jellyfish (v.2.3.0) by counting distinct 31-mers in the reference libraries, with each k-mer in a read mapped to the lowest common ancestor of all reference genomes with exact k-mer matches. Thereafter, each query was classified to a specific taxon with the highest total k-mer hits matched by pruning the general taxonomic trees affiliated with the mapped genomes. Bracken (v.2.5.0) was used to accurately estimate taxonomic abundance, especially at the species level, based on Kraken 2. The read counts of species were converted into relative abundance for further analysis. Linear discriminant analysis Effect Size (LEfSe) was analyzed to identify differential species between groups (LDA score >2, and P-value < 0.05) based on the species profiling.

## Patient tumor RNAseq analysis

The transcriptome data of 3 R and 6 NR patients obtained from above clinical cohort (Baruch et al., 2021) and the RNAseq counts were then normalized through estimating the size factors using DESeq2. Differential gene expression between R and NR tumor samples at post-FMT was performed using DESeq2.

## Gene signature expression and bacterial abundance corrlateion

Spearman’s correlation was used to describe the specific correlation between *Enterococcus lactis* and differential gene as described previously.^91^ Gene signatures were used as following: type I IFN (*CXCL10, IFI16, IFI27, IFI30, IFI6, IFIH1, IFIT1, IFIT2, IFIT3, IFITM1, IFITM2, IFITM3, IFNA1, IFNA2, IFNA4, IFNAR1,IFNAR2, IFNB1, IFNE, IFNW1, IRF1, IRF2, IRF3, IRF5, IRF7, IRF9, ISG15, ISG20, JAK1, JAK2, OAS1, OAS2, SOCS1, STAT1,STAT2, STAT3, TMEM173, TYK2*), dendritic cells (*CD1C, CD1A, LY75, LTB, CD86, FLT3, CD80, CD40, SIRPA*) and CD8 T cells (*CD8A, CD8B, CD3E*).

## Toxicity study

The toxicity study evaluation was provided by a third-party company, Zhaoyan (Suzhou) New Drug Research Center Co., LTD. The objective was to observe the possible adverse effects upon a 28-day dosed period and delayed toxicity following a 28-day recovery period. Briefly, Sprague Dawley (SD) rats (SPF grade, 80 males, and 80 females) were randomly divided into four groups (20 animals per sex) according to body weight. MNC-168 fermental powder was administered to SD rats at doses of 2 × 10.^9^, 1 × 10.^10^, and 5 × 10.^10^ CFU/animal/day, respectively, 0 CFU as negative control, via oral gavage for 28 consecutive days to observe the reversibility of these adverse effects and possibly delayed toxicity following a 28-day recovery period. Body weight and food consumption were measured during the study. Clinical pathology examinations including hematology and coagulation were conducted at the end of the dosing period (Day 29) and at the end of the recovery period (Day 57). Necropsy was conducted after the blood collection of clinical pathology. The corresponding tissues were collected, weighed, preserved, and microscopically examined. After the experiment, the data process and statistical analysis of the corresponding test indicators were performed based on the comparison of the test article treatments with negative control.

## Statistical analysis

Sample sizes and statistical methods are provided in Fig. legends. Statistical analyses were performed using GraphPad software (version 10.2.3) was analyzed for normal distribution prior to comparisons. Statistical significance between two groups was determined using two-tailed unpaired Student’s t test (parametric). For the comparison of multiple groups, statistical significance was determined by one-way ANOVA followed by Tukey’s post hoc test (parametric). For tumor growth measurements, two-way ANOVA were used. The observed differences were deemed statistically significant when the *p* value was less than 0.05. All studies are representative of two or more independent experiments, unless indicated otherwise.

## Data and materials availability

Patient metagenomic sequencing data is previously published and publicly available as Bioprojects PRJEB22863, PRJNA399742, PRJNA678737. Patient tumor transcriptomic data are previously published and publicly available from NCBI’s Gene Expression Omnibus (GEO) under BioProject ID GSE162436. Anminal metabolite data and transcriptomic data are publicly available on FigShare (DOI 1 0.6084/m9.figshare.27887325).

## Supplementary Material

Supplementary file 2.xlsx

Supplementary file 4.xlsx

Supplementary file 3.xlsx

Supplementary file 7.xlsx

Supplementary file 6 .xlsx

Supplementary_Materials R3.docx

Supplementary file 1.xlsx

Supplementary file 5.xlsx
