# Supplementary material for: Live biotherapeutic enterococcus lactis MNC-168 promotes the efficacy of immune checkpoint blockade in cancer therapy by activating STING pathway via bacterial membrane vesicles
Source: Gut Microbes. 2025 Sep 12;17(1):2557978. doi: 10.1080/19490976.2025.2557978 (PMC12439585; doi:10.1080/19490976.2025.2557978)
Supplement: Supplementary_Materials R3.docx [file KGMI_A_2557978_SM1831.docx]

Supplementary Materials for

Live Biotherapeutic *Enterococcus lactis* MNC-168 Promotes the Efficacy of Immune Checkpoint Blockade in Cancer Therapy by Activating STING Pathway via Bacterial Membrane Vesicles

Yibo Xian^1^, Zhipeng Chen^1^, Zhou Lan^1^, Chenchen Zhang^1^, Hao Sun^2^, Zhenzhen Liu^1^, Ping Kong^1^, Yajun Liang^1^, Yingying Zhao^1^, Si-yang Maggie Liu^2^, Yiqi Zhou^1^, Linchuan Gan^1^, Baoxia Li^1^, Xue Su^1^, Baojia Huang^1^, Chen Xiao^1^, Ruijuan Zhu^1^, Guozhen Zhao^1^, Canshan Lao^1^, Chuan-Sheng Lin^1^, Dongya Zhang^1^*, Xianzhi Jiang^1^*

1. Moon (Guangzhou) Biotech Co. Ltd., Huangpu District, Guangzhou, Guangdong, 510530, China

2. Guangdong Lung Cancer Institute, Guangdong Provincial People's Hospital (Guangdong Academy of Medical Sciences), Southern Medical University, Guangzhou, China

These authors contributed equally: Yibo Xian and Zhipeng Chen

Correspondence to: Xianzhi Jiang, Email: [jxz@moonbio.com](mailto:jxz@moonbio.com); Dongya Zhang, Email: [zhangdy@moonbio.com](mailto:zhangdy@moonbio.com)

**This PDF file includes:**

Materials and Methods

Supplementary Figures

Figures. S1 to S15

**Other Supplementary Materials for this manuscript include the following:**

Flie S1 to S7

Materials and Methods

**Bacteria**

*Enterococcus spp*. strains including MNC-168 were supplied by Moon (Guangzhou) Biotech Co.,Ltd. that isolated from healthy human donors. *Enterococcus lactis* MNC-168 was deposited under GDMCC NO. 61121 in Guangdong Microbial Culture Collection Center at Guangdong Institute of Microbiology. All *Enterococcus spp*. strains were inoculated into self-optimized medium MM01 (peptone, 15 g; glucose, 20 g; yeast extract, 15 g; cysteine, 1 g; sodium acetate, 5 g; sodium citrate, 4 g; dipotassium phosphate, 2 g; magnesium sulfate, 0.1 g; manganese sulfate, 0.05 g; Tween 80, 1 g contained per liter; pH 6.3-6.5) and incubated under anaerobic conditions at 37 ℃ for 24 hours. All processing of bacteria was performed in an anaerobic platform and all the reagents were vented in an anaerobic atmosphere for at least 24 hours prior to use.

**Microbiome enrichment analysis between patients with response and non-response to immunotherapy**

The data for analyzing the microbiome enrichment between response and non-response to immunotherapy was origin from the clinical cohort (Routy et al., 2017: n=249 patients; Matson et al., 2018: n=42 melanoma patients). Key confounders were accounted in these studies including antibiotic use, detailed patient histories. Alpha diversity was calculated to estimate the species richness of a sample using Shannon index and Simpson index, which were calculated using R package ‘vegan’ (ordination methods, diversity analysis and other functions for community and vegetation ecologists), based on species relative abundance. Beta diversity was calculated, using sample-sample distance (including the Jensen-Shannon distance and the Bray distance), to estimate the difference of the species richness between different samples based on species relative abundance. Bacterial taxonomic comparisons were estimated using Wilcoxon rank sum test between R and NR groups based on the species relative abundance, and the significantly different species were determined by Wilcoxon rank sum test (p<0.05).

**Genomic similarity, virulence factors and resistance gene analysis of *Enterococcus. Spp.* between clinical clinical source and our isolated strain**

Genomes of clinical strains were download from NCBI Pathogen Detection (genus limited to “Enterococcus”, isolation source limited to “hospital”, isolation type limited to “clinical”). The average nucleotide identity (ANI) analysis among strains in this study and clinical strains was conduct by fastANI (Version 1.33). Genomes of clinical strains were then analyzed for genetic coding by the same method used in this study. Virulence factors were identified by NCBI blastp ( Version: 2.7.1+, Database: VFDB, updated on 2019/09/19 ). Antibiotic Resistance was identified by RGI pipline ( Version: 6.0.4 ).

**Cell culture**

The MC38 and H22 cells were purchased from Nanjing Kebai Biotechnology. The Renca, WEHI164, JC, CT26, 293T and THP-1 cells were purchased from the American Type Culture Collection (ATCC). PBMCs were purchased from Milestone® Biotechnologies. All cell lines were authenticated using DNA fingerprint analysis and were negative for mycoplasma contamination. MC38 and 293T cells were cultured in DMEM medium while other cells were cultured in RPMI-1640 medium supplemented with 10% fetal bovine serum (Gibco) and 100 units per mL of penicillin (Gibco) and streptomycin (Gibco). All cells were cultured at 37 ℃ with 5% CO_2_ atmosphere.

**Mice**

C57B6/J (male) and BALB/c-Nude (male) mice were purchased from GemPharmatech Inc. BALB/c (male) mice were purchased from Shanghai Model Organisms Center Inc. All the mice were at the age of 6-8 weeks old and maintained in a specific pathogen-free environment. All animal experiments were conducted in accordance with protocols approved by the Institutional Animal Care and Use Committee of Moon (Guangzhou) Biotech Co.,Ltd., in compliance with the Guide for the Care and Use of Laboratory Animals. The number of mice in this study provide sufficient statistical results, including for tumor size, cytokines/chemokines and immune profiling. All the mice were randomly distributed into the experimental groups for this study.

**Syngeneic tumor mouse model**

For MC38 tumor model, C57B6/J male mice were subcutaneously (s.c.) injected with 2×10^4^ MC38 colon cancer cells and randomly divided into control group and MNC-168 group which were respectively intragastric (i.g.) administrated with control medium or 2×10^9^ CFU MNC-168 every day following tumor cells inoculation. When the tumor volume was around 50-100 mm^3^, mice of control group and MNC-168 group were intraperitoneally (i.p.) injected with or without 10 mg/kg anti-PD-1 monoclonal antibody (mAb; clone RMP1-14, BioXCell) in 0.9% sodium chloride every 3 days for 4-6 times in total. Tumor size was measured every two days until the endpoint. For dose assessment in antitumor effect, 2×10^8^ CFU, 2×10^9^ CFU and 2×10^10^ CFU of MNC-168 were administrated in MC38 tumor model with or without anti-PD-1, respectively. Further, for evaluating the sole role of MNC-168 in anti-tumor, gut microbes were depleted with antibiotic cocktail (ATB including 1 mg/mL ampicillin, 5 mg/mL streptomycin, 1 mg/mL colistin) by supplementing the mice with ATB containing water two weeks before administration of MNC-168 with or without anti-PD-1 in MC38 tumor model. For multiple tumor models, BALB/c mice were subcutaneously injected with 5×10^4^ Renca, 5×10^4^ WEHI164, and 5×10^4^ H22 cells, respectively, to establish diverse syngeneic tumor model and administrated with MNC-168 or combined with anti-PD-1 similar to MC38 tumor model. For comparison of the anti-tumor effect of MNC-168 between immunocompetent and T cell deficient mice, BALB/c and nude mice were subcutaneously injected with 1×10^6^ CT26 cells, respectively. MNC-168 (2×10^9^ CFU) or combined anti-PD-1 (10 mg/ kg) were administrated via intragastrically and intraperitoneally injection, respectively.

**CD34^+^ HSC immune system humanized mouse model**

Humanized female mice reconstituted with CD34+ hematopoietic stem cells (HSCs) and exhibiting hCD45+ cell engraftment exceeding 25% at approximately 16 weeks post-transplantation were subcutaneously inoculated with 5×10^6^ HCC827 cells. The anti-tumor efficacy of MNC-168, either as a monotherapy or in combination with anti-PD-1 antibody, was subsequently evaluated.

**Bacterial colonization analysis**

Mouse feces were collected at the endpoint of the MC-38 study. Total DNA was extracted using Fecal genome DNA extraction kit (TIANGEN, DP328) according to the manufacturer’s instructions. DNA concentrations were measured by Microplate reader. Reference plasmid containing the MNC-168 gene was subjected to a 10-fold dilution gradient for the purpose of generating a standard curve. Absolute quantification of MNC-168 were performed using real-time PCR (Applied Biosystems Quantstudio3). For total bacteria analysis, primers were used as follows: Total-F- CGGYCCAGACTCCTACGGG; Total-R- TTACCGCGGCTGCTGGCAC. For MNC-168 analysis, primer sequences were as follows: 168F-AACTTGTGTCGTATCCCTTTGTCA; 168R-TAACCCGAAAGTTTCTGATAAAGATG; Probe: TTATGCGGAAAGAAGAT-MGB-NFQ. Quantification was calculated according to the standard curve and the CT value of the sample. The content unit of MNC-168 in the final calculated fecal sample was uniformly converted to CFU/g.

**Flow cytometry analysis**

Tumor, spleen, and MLN from the mice were collected at the endpoint of the MC-38 study. Dissected tumors were cut into small pieces, homogenized using tissue crusher, and digested in digestion buffer (containing 1 mg/mL collagenase IV, 20 µg/mL DNase I and 10% FBS) using shaker with 200 RPM at 37 ℃ for 20 min and then filtered through a 70 μm cell strainer. The spleens and MLNs were mashed in PBS and filtered through a 70 μm cell strainer. Single cell suspensions of tumor, spleen and MLN were incubated with anti-mouse CD16/CD32 (BD, 553142) to block the Fc receptor. Cells viability was stained with LIVE/DEAD Fixable Dead Cell Stain kit (Thermofisher, L3224) followed by staining with the corresponding antibodies. The following anti-mouse antibodies were used: CD45-AF700 (BioLegend, 103127), CD3-APCCY7 (BioLegend, 100222), CD4-APC (BioLegend, 100516), CD8-PC5.5 (BioLegend, 100734), CD45-PC5.5 (BioLegend, 103132), CD4-PE (BioLegend, 100512), CD45-PECY7 (Thermofisher, 25-0451-82), CD11c-PC5.5 (BioLegned, 117328), MHCII-APC (BioLegned, 107614), IFN-γ-PECY7 (BioLegend, 505826), TNF-α-PE (BD, 554419), Foxp3-APC (BD, 560401). For intracellular markers, cells were fixed and permeabilized using Cytofix/Cytoperm™ Fixation/Permeabilization buffer (BD, 554714) or FOXP3/Transcription Factor Staining Buffer kit (Thermofisher, 00-5523-00) according to the manufacturer’s instructions after surface staining and incubated with the corresponding antibodies.

The stained cell analysis was performed with DxFLEX (Backman) and the data were analysis with CytExpert software (version 2.0.0.283). The preliminary gating strategy for all analysis is: (1) FSC-A versus SSC-A for debris exclusion; (2) FSC-H versus FSC-A for doublet exclusion; (3) SSC-A versus Live/Dead for dead cell exclusion; (4) SSC-A versus CD45 for positive cells (leukocytes); (5) CD11c versus CD45 for DCs, while CD11c versus MHC II from CD45^+^ cells for DC MHC II positive analysis; (6) CD3 versus CD45 for T cells; (7) CD4 versus CD8 from CD3^+^CD45^+^ cells for CD4^+^ and CD8^+^ T cells, while IFN-γversus CD4 or CD8 from CD3^+^CD4^+^ or CD3^+^CD8^+^ T cells for IFN-γ^+^CD4^+^ or IFN-γ^+^CD8^+^ analysis; (8) Foxp3 versus CD3 from CD45^+^ cells for Treg cells.

**Cytometric Bead Array (CBA)**

Supernatant of THP-1 or PBMC co-cultured with MNC-168 were collected and analyzed with CBA Kit, including IL-6 (BD, 558276), TNF-α (BD, 560112), IL-1β (BD, 558279), CCL5 (BD, 558324), CXCL10 (BD, 558280) and MCP-1 (BD, 558287). For mouse, blood from the mice eye socket vein of mice were collected in EDTA anticoagulant tube and centrifuged with 500× g, 10 min for plasma. Cytokines of mouse plasma were analyzed by a LEGEND plex Custom Mouse Panel Kit, including IFN-γ, IL-2, IL-1β, CXCL10(IP10), CXCL1(KC), TNF-α, CXCL9(MIG) and IL-6 (BioLegend, US, CLPX-200511OY-GENEMA).

**Immunohistochemistry**

For histologic analysis, tumor specimens of H22 mouse model were fixed with 4% paraformaldehyde, dehydrated in ethanol, embedded with paraffin, and stained with hematoxylin and eosin (H&E) and stained with CD4 (SouthernBiotech, 1540-01), CD8 (Servicebio, GB13429), IFN-γ (BioLegend, 606853) and Foxp3 (BioLegend, 126406) antibodies, respectively. Image acquisition was using Panoramic section scanner (Hungary, 3DHISTECH). Positive stained cells in an area of the slide were analyzed and calculated using Halo (v3.0.311.314) software with Indica labs - Multiplex IHC v2.2.0 module Sample preparation and image acquisition were conducted by Wuhan servicebio technology CO.,LTD. The quantification and graphical representation of data were conducted using GraphPad software (version 8.0.1).

**Transcriptomic profiling of tumor**

At the endpoint of treatment model, tumors were collected, and the total RNA was extracted with MiniBEST Universal RNA Extraction Kit (Takara, 9767) following by constructing the library for transcriptome sequencing. The follow process of RNA-sequencing was conducted by Novogene (Beijing, China). For analysis, briefly, RNA-sequencing reads were filtered to obtain high-quality reads using fastp (version 0.20.0) software. This process includes removing sequencing adapters, primer dimers, sequences which containing N bases > 5 and trimming bases with a quality score < Q15. Then, using HISAT2 (version 2.2.1) software, trimmed reads were mapped to GRCm38 (Ensembl release 101) reference genome. For the differentially expressed genes (DEGs) enrichment analysis, the featureCounts function (version 2.0.1) was used to count transcript features from all samples, and sum the counts to the gene level. Then, differential expressed genes (DEGs) between each group were calculated by the DESeq2 R package. Subsequently, Gene Ontology (GO) and Gene set enrichment analysis (GSEA) was performed using the cluster Profiler based on the gene set from org.Mm.eg.db.

**Metabolomic profiling of cecal content**

Metabolomic profiling was conducted by Novogene (Beijing, China). Briefly, metabolites were extracted from cecal content with prechilled 80% methanol and 0.1% formic acid and further clean with 60% methanol LC-MS grade water following injected into the LC-MS/MS system analysis. UHPLC-MS/MS analyses were performed using a Vanquish UHPLC system (ThermoFisher, Germany) coupled with an Orbitrap Q ExactiveTM HF mass spectrometer (Thermo Fisher, Germany) in Novogene Co., Ltd. (Beijing, China). For metabolite identification, the raw data were processed using the Compound Discoverer 3.1 (CD3.1, ThermoFisher). The normalized data was used to predict the molecular formula, and the peaks were matched with the mzCloud (<https://www.mzcloud.org/>), mzVault and MassList database. Statistical analyses were performed using the statistical software R (R version R-3.4.3), Python (Python 2.7.6 version) and CentOS (CentOS release 6.6). These metabolites were annotated using the KEGG database (https://www.genome.jp/kegg/pathway.html), HMDB database (https://hmdb.ca/metabolites) and LIPIDMaps database (http://www.lipidmaps.org/). The univariate analysis (t-test) was used to calculate the statistical significance (P-value). The metabolites with VIP > 1 and P-value< 0.05 and fold change≥2 or FC≤0.5 were considered to be differential metabolites. The correlation between differential metabolites were analyzed by cor in R language (method=pearson).

**16S rRNA sequencing**

Fresh fecal pellets collected from individual mice were stored at – 80℃ until analysis. DNA extraction was performed by Fecal genome extraction kit (Tiangen, DP328), which followed by 16S ribosomal RNA (rRNA) gene amplicon sequencing at the Novogene (Beijing, China) using their standard workflow. Samples were sequenced on the Novaseq 6000 platforms.

For analysis, briefly, paired-end reads were filtered by fastp software to obtain high-quality reads and were merged using bwa pemerge function. High quality reads were further analyzed using the Quantitative Insights Into Microbial Ecology (QIIME2 version 2019.7) bioinformatics pipeline. In our study, DADA2 was utilized to generate amplicon sequence variants (ASVs) by denoising, removing chimeric and short reads. Furthermore, SILVA v138_99 16S rRNA gene database were used to train Naïve Bayesian classifier for taxonomic classification of ASVs. The biodiversity of the samples, including alpha and beta diversity, was calculated by using the q2-diversity plugin. Finally, the linear discriminant analysis (LDA) effective size (LefSe) was conducted to identify the significantly different species.

**Plasmids**

For reporter gene plasmid construction, the NF-κB reporter gene and STING reporter gene were acquired by referring the reporter plasmid 4x NFκB Luc (Addgene, Plasmid #111216) and IFN-Beta_pGL3 (Addgene, Plasmid #102597), respectively. The reporter genes were subcloned into a lentivirus plasmid pCMV-Puro which deleted the original CMV promoter to construct the lentivirus reporter plasmid (pNFκB-Luc-puro and pSTING-Luc-puro).

**Reporter gene cell engineering**

Lentiviruses containing NFκB-Luc or STING-Luc reporter genes were produced in 293T cells through co-transfection of pNFκB-Luc-puro or pSTING-Luc-puro with psPAX2 and pMD2.G plasmids. To generate THP-1-NFκB-Luc or THP-1-STING-Luc cell lines, THP-1 cells were infected with the above packaged lentiviruses and the stable clone cell line was selected by puromycin (2 µg/mL).

**RNA interference**

THP-1-IFN-β-reporter cells were seeded in 24 wells plate, two distant siRNA fragment (50 µM) of TBK1 or IRF3 were transfected with Lipofectamine™ RNAiMAX (Thermo fisher, 13778100). Post 24 hours transfection, cells were used for RNA knockdown efficiency detection or downstream experiments. SiRNA fragments were purchased from RIBOBIO, China. Sequences of the siRNA, siTBK1-1#: CCACAAATTTGATAAGCAA; siTBK1-2#: GAAGAAATATGGAGCAACA; siIRF3-1#: GTGGACCTGCACATTTCCA; siIRF3-2#: AGACATTCTGGATGAGTTA.

**Reporter gene activity assay**

THP-1-NFκB-Luc or THP-1-STING-Luc cells were seeded in white flat 96 well plate at 1×10^5^ cell/well. After 24 h treatment with MNC-168 supernatant or BMVs, the fluorescence detection was performed according to the manufacturer’s instructions of Luminescent Kit (Promega, E2610) on the Microplate Reader.

**RT-qPCR analysis**

Total RNA was extracted using MiniBEST Universal RNA Extraction Kit (Takara, 9767) following the instruction manual. For reverse-transcription, 2 μg total RNA was reverse-transcribed to cDNA with PrimeScript™ RT reagent Kit with gDNA Eraser (Takara, RR047A). Human GAPDH gene was used as an internal control. Real-time PCR was performed with Applied Biosystems Quantstudio3 Real-time PCR machine and PrimeScript RT Enzyme Mix reagent (Takara, RR037Q) with the following primers:

qh-IL1β-F：CCACAGACCTTCCAGGAGAATG

qh-IL1β-R：GTGCAGTTCAGTGATCGTACAGG

qh-TNFα-F：GAGGCCAAGCCCTGGTATG

qh-TNFα-R：GGGCCGATTGATCTCAGC

qh-IFNβ-F：ATGACCAACAAGTGTCTCCTCC

qh-IFNβ-R：GGAATCCAAGCAAGTTGTAGCTC

qh-GAPDH-F：ACAACTTTGGTATCGTGGAAGG

qh-GAPDH-R：GCCATCACGCCACAGTTTC

qH-TBK1-F：CAACCTGGAAGCGGCAGAGTTA

qH-TBK1-R：ACCTGGAGATAATCTGCTGTCGA

qH-IRF3-F：TCTGCCCTCAACCGCAAAGAAG

qH-IRF3-R：TACTGCCTCCACCATTGGTGTC

**Western bolting**

Total protein of the cells was extracted with RIPA lysis buffer containing protease inhibitor cocktail and phosphatase inhibitor cocktail (Yeasen, 20214ES03, 20109ES05). Protein quantification was detected by the BCA (Beyotime, P0012). Equal amounts of protein were separated by SDS-PAGE and transferred to PVDF membrane (Millipore). Membranes were blocked in 5% BSA and incubated with relevant primary antibodies at 4℃ overnight. The membrane were washed with TBST and incubated with secondary HRP antibodies. Next, the membrane was then washed with TBST. ECL was applied for film development. Antibodies were used as follows: Phospho-TBK1/NAK (Ser172) (D52C2) XP® Rabbit mAb (CST, 5483), TBK1/NAK (D1B4) Rabbit mAb (CST, 3504), Phospho-IRF-3 (Ser386) (E7J8G) XP® Rabbit mAb (CST, 37829), IRF-3 (D6I4C) XP® Rabbit mAb (CST, 11904), β-actin Rabbit mAb (SDT-R015) (STARTER, S0B0005) Goat anti-Rabbit IgG(H+L), HRP (STARTER, S0B4002).

**ELISA assay**

For mouse IFN-β assay, serum from the mouse tumor model were collected and the concerntraion of IFN-β was measured with Mouse Interferon β,IFN-β/IFNB ELISA Kit (CUSABIO, CSB-E04945m). For cell supernatant assay, cell culture medium was collected and measured with Human Interferon β,IFN-β/IFNB ELISA Kit (CUSABIO, CSB-E09889h).

**Bacterial membrane vesicles isolation**

Bacterial MVs were isolated from MNC-168 culture medium by commercial Bacterial MVs isolation kit (Rengen Biosciences, BacMV10-10). Briefly, 10 mL of culture medium was fully mixed with 1 mL Bind buffer. Then 400 µL Binding Resin was added into the above mixture and mix upside down at room temperature for 15 min. Centrifuge the above mixture at 1,500× g, for 2 min. Discard supernatant and transfer the resin to the purification column and wash the MVs with washing buffer for 2 times. Finally, MVs were eluted by Elution buffer. The concentrated bacterial MVs could be used for further experiments or stored at -80 ℃.

**Fecal membrane vesicles DNA extraction and Metagenomic sequencing**

The faecal membrane vesicles were isolated and lysed, followed by extraction of vesicle DNA through isopropyl alcohol precipitation. Subsequently, the concentration and quality of DNA were determined using Qubit 4.0 and NanoDrop spectrophotometer, respectively, while the integrity of DNA was assessed through 1% agarose electrophoresis. The library was generated by fragmenting the DNA and ligating adapters, followed by target fragment enrichment. The size and concentration of fragments were measured using Qseq400 and Qubit 4.0, respectively. Finally, Illumina NextSeq 2000 was employed for DNA sequencing.

**Bacterial membrane vesicles abundance analysis**

Metagenomic data was analyzed with Kaiju and the representative genomes of bacteria, archaea, and viruses in NCBI RefSeq as references, conducted a relative abundance analysis comparing the MNC-168 treated group to the control group. Specifically, compared the relative abundance of Enterococcus between these two groups. DiTASiC was employed to analyze the level of abundance for different strains within Enterococcus.

**Nanosight measurement**

BMVs size was measured by the NanoSight NS300 analyzer (Malvern Instruments Ltd, Malvern, UK). Briefly, isolated MVs were diluted 200 times with PBS, and the size, distribution, and concentration of MVs was analyzed with NanoSight NS300 combined with the software NTA 3.4 Build 3.4.003.

**Transmission electron microscopy (TEM)**

BMVs morphology observation was performed by TEM using negative staining. Briefly, 5 μL of isolated MVs was deposited onto copper mesh. After incubation for 1 min, they were stained with 1% phosphotungstic acid for 2 min, rinsed with pure water for twice, air-dried at room temperature for 10 min, and subsequently examined under transmission electron microscopy (Tecnai G2 Spirit Bio TWIN) with 80 kV voltage.

**BMVs DNA electrophoresis**

BMVs of MNC-168 were lysed using DNA loading buffer (NEB, B7024) at 95 ℃ for 5 min and subsequently loaded onto a 1% agarose gel for electrophoresis.

**In vivo tracking of MNC-168 MVs**

The fluorescent dye 1, 1’-dioctadecyl-3, 3, 3’, 3’-tetramethylindotricarbocyanine iodide (DiR) (Yeasen, 40757ES25) was used to label MNC-168 MVs. Purified MVs were incubated in the presence of 5 mM DiR for 20 min at 37 ℃, then the MVs were washed with PBS ultrafiltration for 3 times to remove the unbounded dye. Finaly, the labeled MVs were resuspended in PBS prior to use. CT-26 tumor model was built according to the previous method, when the tumor volume reached 400-500m^3^, the labeled MVs (200 ug) were injected intravenously or gaveged to the mouse and monitor the fluorescence in mouse at different timepoint using the IVIS spectrum (Tanon ABL X5). Post 48 hours administration, the tumor-bearing mice were anesthetized with pentobarbital and the fluorescence images of the organ and tumor were captured using IVIS spectrum. For doses injection study, the labeled MVs from 100 ug to 25 ug were injected intravenously and monitor the fluorescence in mouse at different timepoint, which anesthetized post 48 hours for imaging the fluorescence in tumor.

**Patient metagenome analysis**

Metagenome data obtained from 3 R and 7 NR anti-PD-1-refractory metastatic melanoma patients treated with fecal matter transplant (FMT) and reinduction of nivolumab (anti-PD-1), which is a phase 1 clinical trial (NCT03353402), the primary objectives were to assess the safety and feasibility (Baruch et al., 2021). Key confounders were accounted in this study including antibiotic use, dietary control, genetic factors.Raw reads were quality trimmed using fastp (v0.19.7) and filtered against the human genome (hg19) using Bowtie2 (v2.3.4). After quality trimming and filtering, the clean reads were used in downstream analyses. The taxonomic classification of bacteria was assigned to metagenomic reads using Kraken 2 (v.2.1.2), an improved metagenomic taxonomy classifier that utilizes k-mer-based algorithms. A custom database consisting of bacterial reference genomes from the NCBI RefSeq database (accessed in January 2023) was built using Jellyfish (v.2.3.0) by counting distinct 31-mers in the reference libraries, with each k-mer in a read mapped to the lowest common ancestor of all reference genomes with exact k-mer matches. Thereafter, each query was classified to a specific taxon with the highest total k-mer hits matched by pruning the general taxonomic trees affiliated with the mapped genomes. Bracken (v.2.5.0) was used to accurately estimate taxonomic abundance, especially at the species level, based on Kraken 2. The read counts of species were converted into relative abundance for further analysis. Linear discriminant analysis Effect Size (LEfSe) was analyzed to identify differential species between groups (LDA score >2, and P-value< 0.05) based on the species profiling.

**Patient tumor RNAseq analysis**

The transcriptome data of 3 R and 6 NR patients obtained from above clinical cohort (Baruch et al., 2021) and the RNAseq counts were then normalized through estimating the size factors using DESeq2. Differential gene expression between R and NR tumor samples at post-FMT was performed using DESeq2.

**Gene signature expression and bacterial abundance corrlateion**

Spearman’s correlation was used to describe the specific correlation between *Enterococcus lactis* and differential gene as described previously{Karlsson, 2013 #102}. Gene signatures were used as following: type I IFN (*CXCL10, IFI16, IFI27, IFI30, IFI6, IFIH1, IFIT1, IFIT2, IFIT3, IFITM1, IFITM2, IFITM3, IFNA1, IFNA2, IFNA4, IFNAR1,IFNAR2, IFNB1, IFNE, IFNW1, IRF1, IRF2, IRF3, IRF5, IRF7, IRF9, ISG15, ISG20, JAK1, JAK2, OAS1, OAS2, SOCS1, STAT1,STAT2, STAT3, TMEM173, TYK2*), dendritic cells (*CD1C, CD1A, LY75, LTB, CD86, FLT3, CD80, CD40, SIRPA*) and CD8 T cells (*CD8A, CD8B, CD3E*).

**Toxicity study**

The toxicity study evaluation was provided by a third-party company, Zhaoyan (Suzhou) New Drug Research Center Co., LTD. The objective was to observe the possible adverse effects upon a 28-day dosed period and delayed toxicity following a 28-day recovery period. Briefly, Sprague Dawley (SD) rats (SPF grade, 80 males and 80 females) were randomly divided into 4 groups (20 animals per sex) according to body weight. MNC-168 fermental powder was administered to SD rats at doses of 2×10^9^, 1×10^10^, and 5×10^10^ CFU/animal/day, respectively, 0 CFU as negative control, via oral gavage for 28 consecutive days to observe the reversibility of these adverse effects and possible delayed toxicity following a 28-day recovery period. Body weight and food consumption were measured during the study. Clinical pathology examinations including hematology and coagulation were conducted at the end of the dosing period (Day 29) and at the end of the recovery period (Day 57). Necropsy was conducted after the blood collection of clinical pathology. The corresponding tissues were collected, weighed, preserved and microscopically examined. After the experiment, the data process and statistical analysis of the corresponding test indicators were performed based on the comparison of the test article treatments with negative control.

**Statistical analysis**

Sample sizes and statistical methods are provided in the figure legends. Statistical analyses were performed using GraphPad software (version 8.0.1) was analyzed for normal distribution prior to comparisons. Statistical significance between two groups was determined using two-tailed unpaired Student’s t test (parametric). For the comparison of multiple groups, statistical significance was determined by one-way ANOVA followed by Tukey’s post hoc test (parametric). For tumor growth measurements, two-way ANOVA were used. The observed differences were deemed statistically significant when the P value was less than 0.05. All studies are representative of two or more independent experiments, unless indicated otherwise.

Supplementary figures


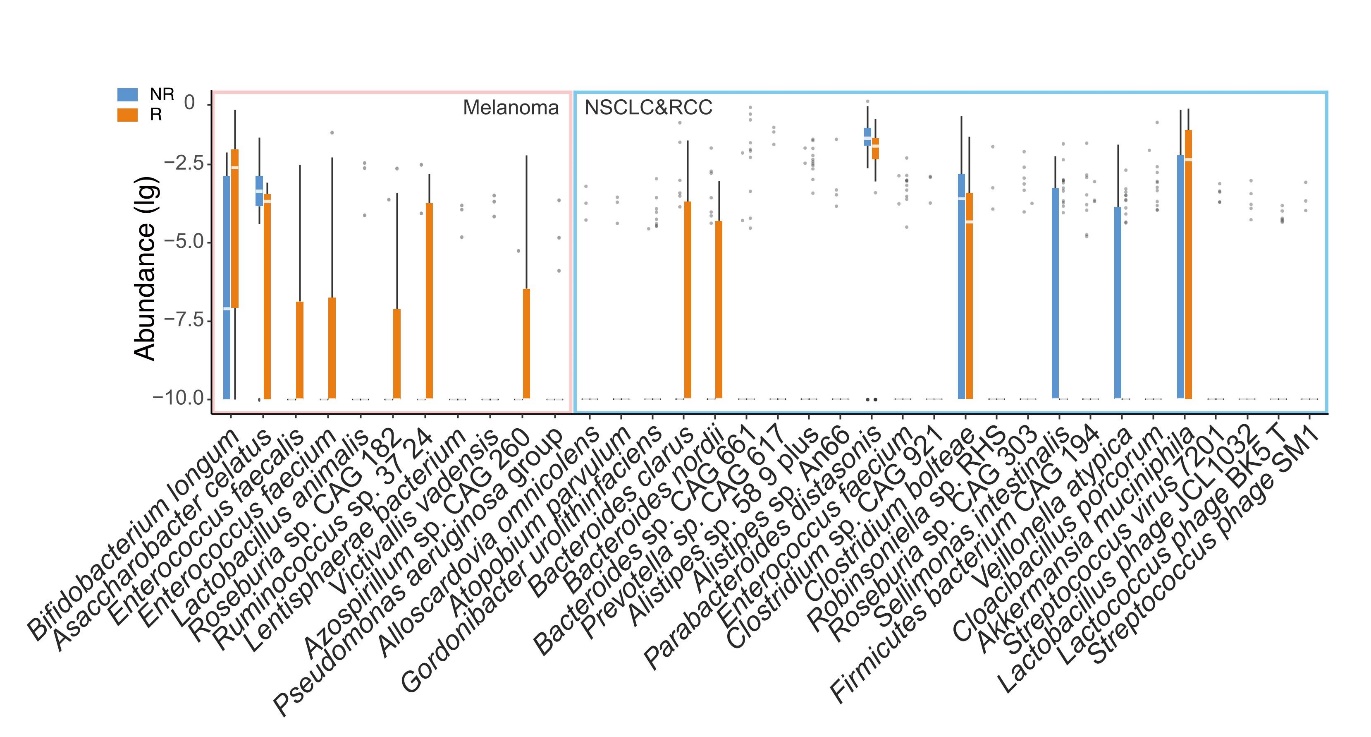


Figure. S1.

Differential abundance analysis of gut microbes in melanoma and NSCLC/RCC patients with response and non-response to immunotherapy


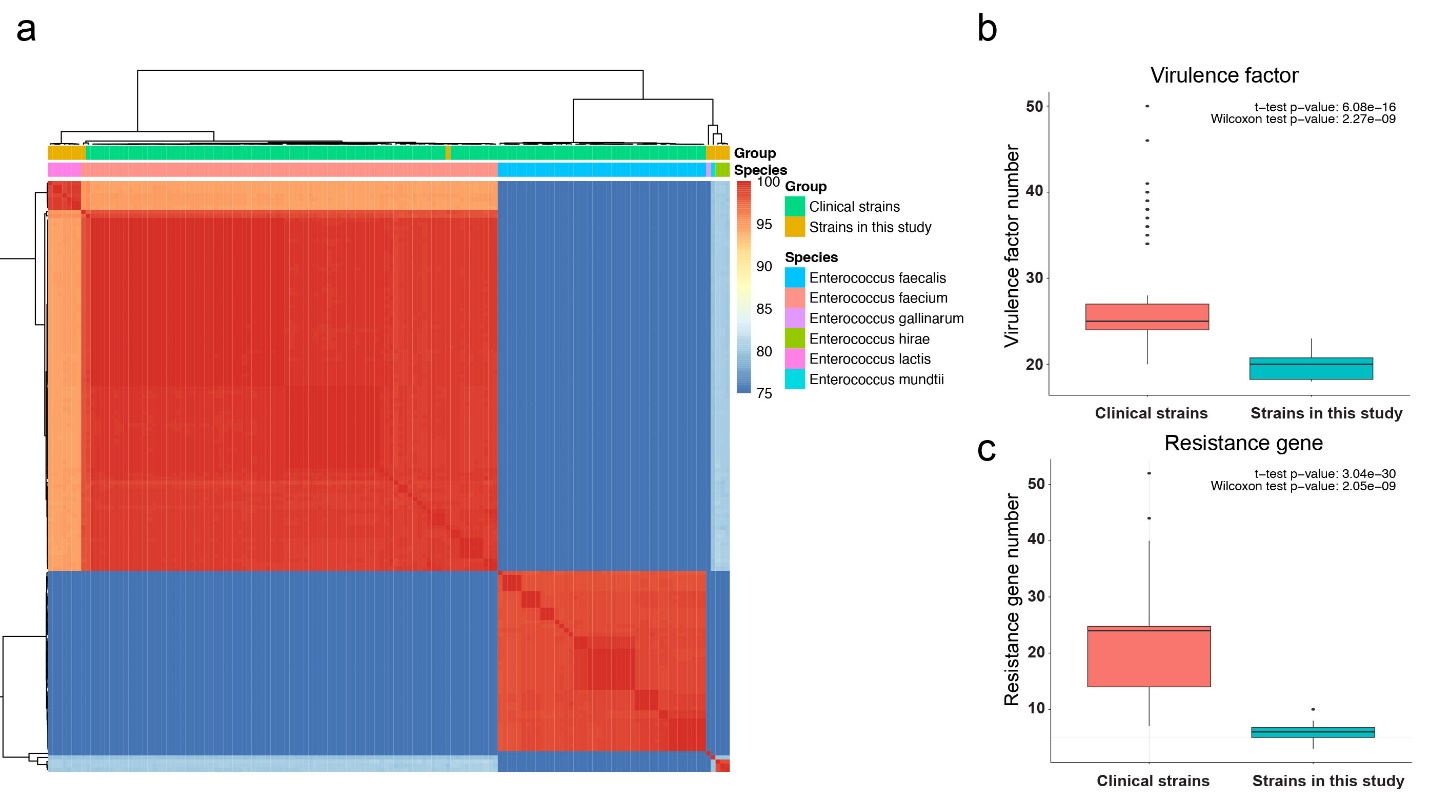


**Figure. S2.**

Genomic similarity, virulence factor and resistance gene analysis of the *Enterococcus.spp.* between our isolated strains and clinical strains. a. Genomic similarity analysis. b. Virulence factor comparison. c. Resistance gene comparison.


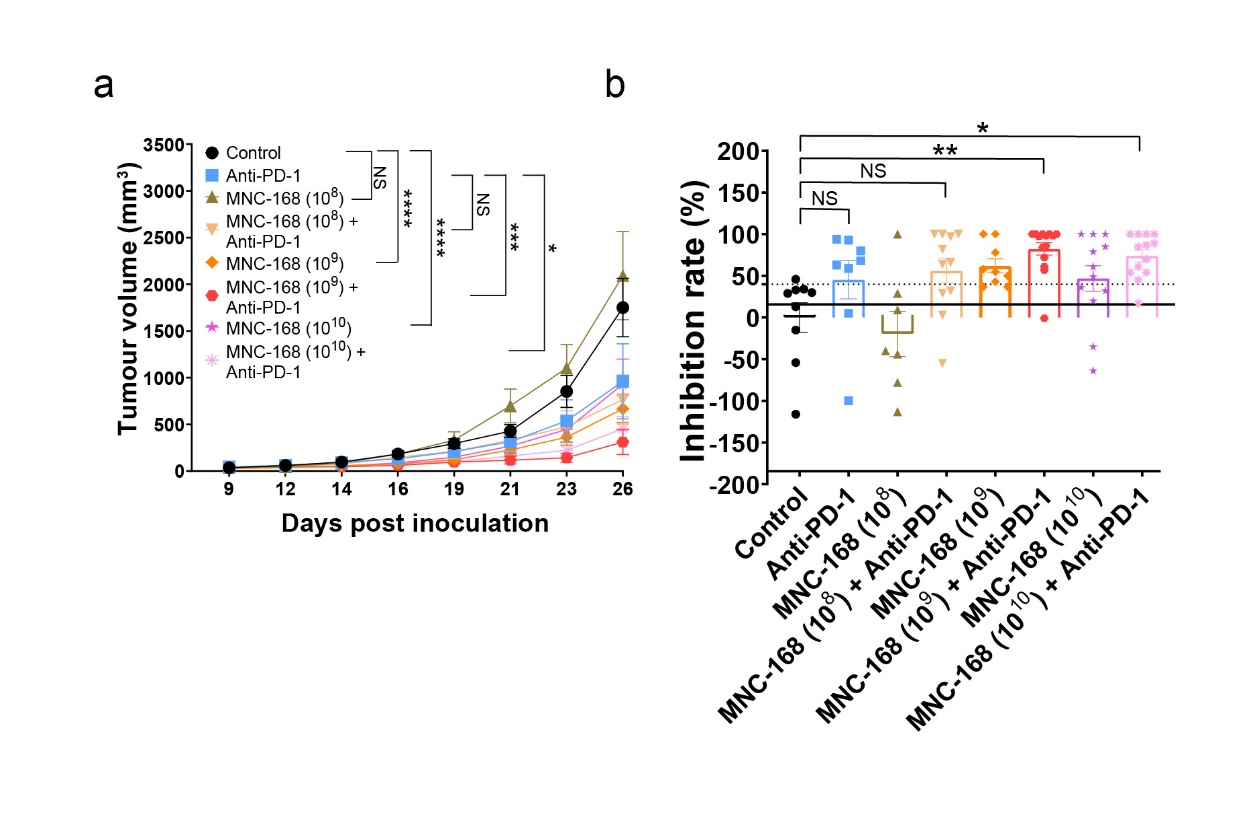


**Figure. S3.**

Assessment of the relationship between Dose and Drug Efficacy. a. Tumor growth curves showing the changes in tumor volume after administration of different doses of MNC-168, with or without anti-PD-1. Mice per group n ≥ 7. b. Tumor growth inhibition rate analysis with indicated group on Day 26.


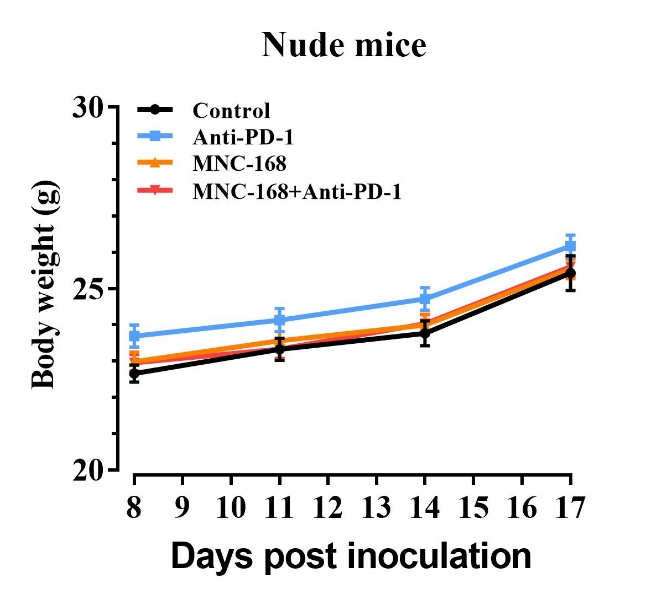


**Figure. S4.**

Body weight growth curve of the nude mice upon MNC-168 daily treatment


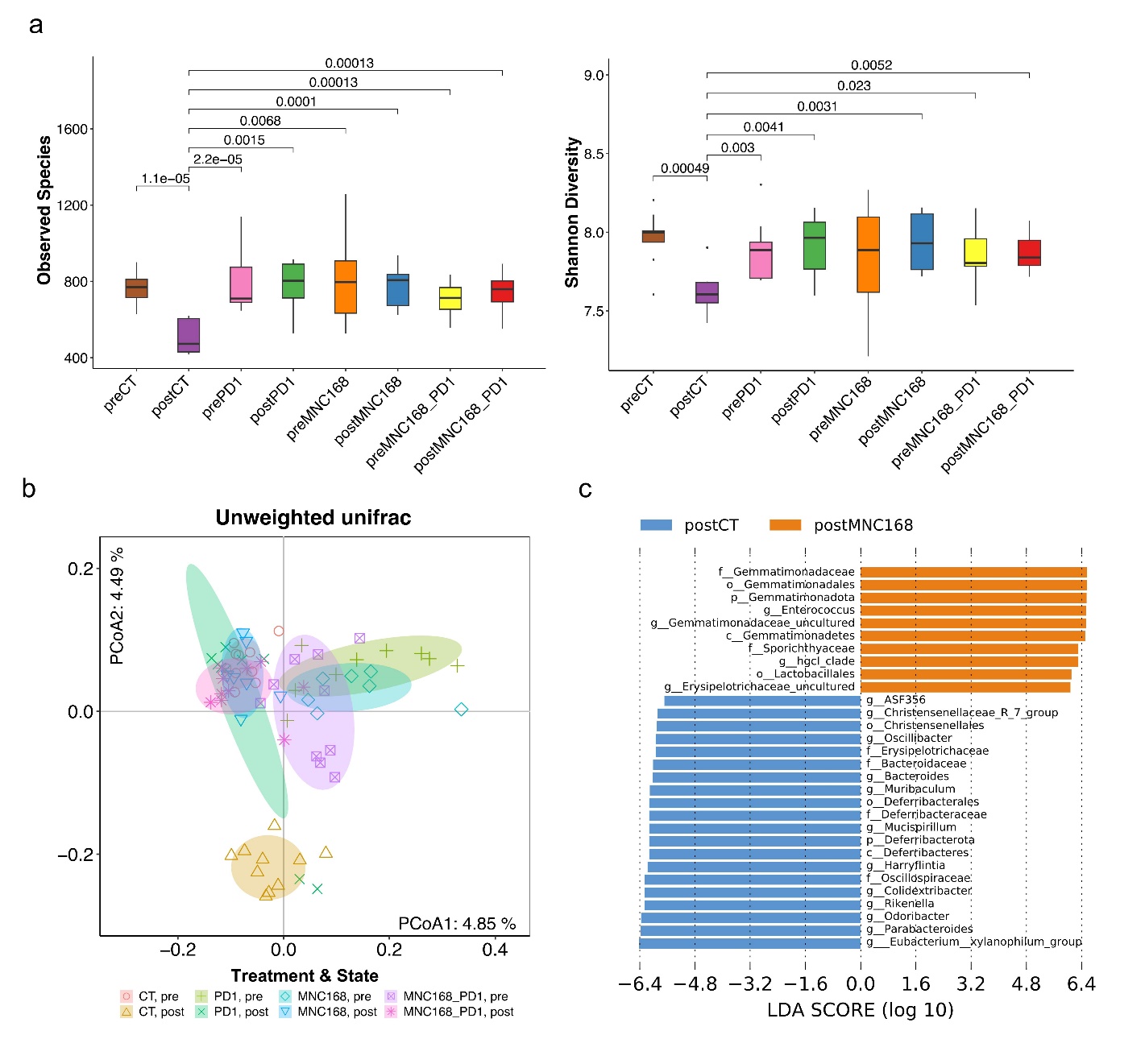


Figure. S5.

Influence of MNC-168 on gut microbiota in syngeneic tumor model treated with MNC-168. a. The analysis of gut microbiota abundance and diversity in a syngeneic tumor model following the indicated treatment. b. PCA analysis of the gut microbiota. c. LEfSe analysis was conducted to determine the bacteria most likely to explain differences between control and post MNC-168 treatment groups.


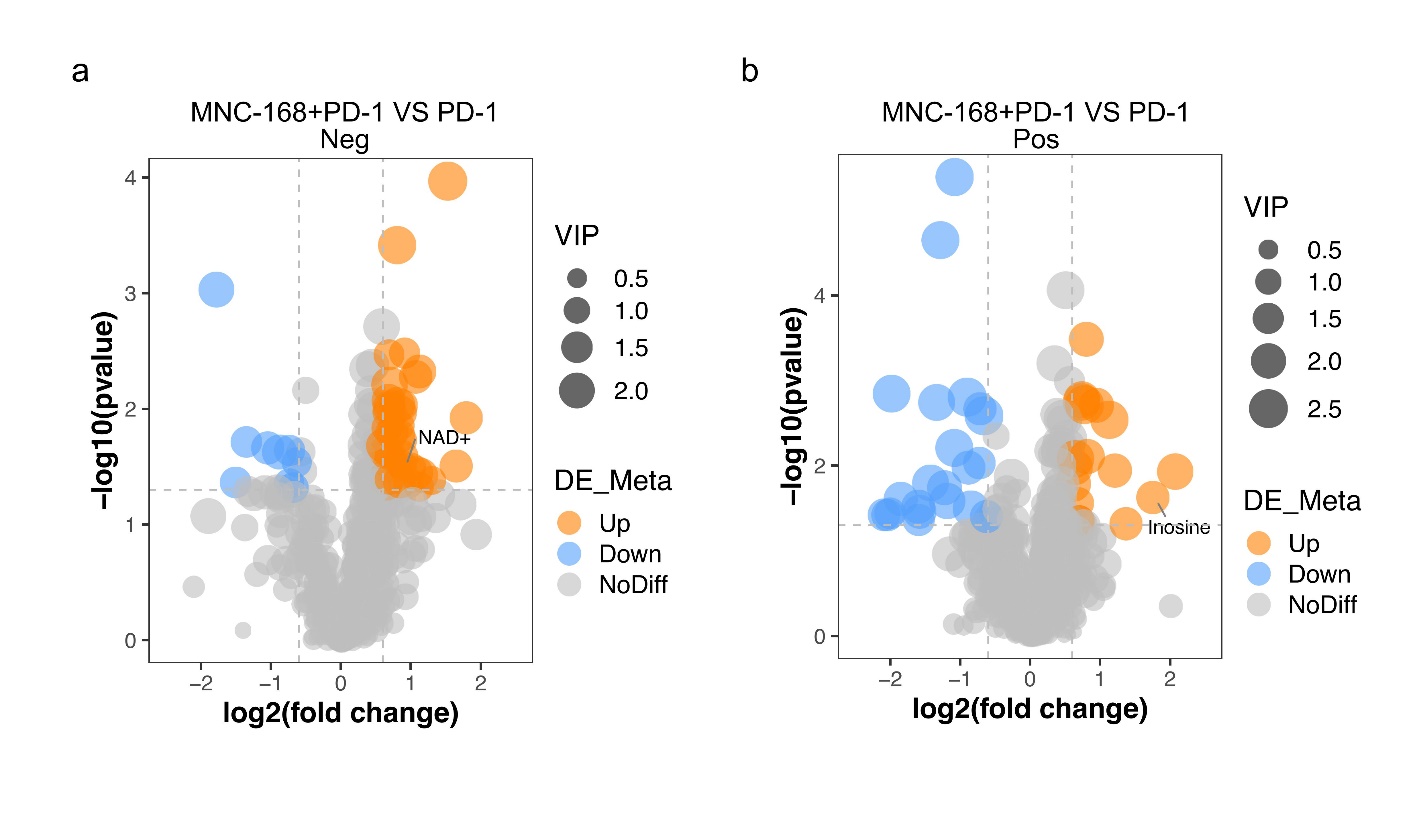


Figure. S6.

Metabolomics profiling of cecal content. a-b. Volcano plot comparing the differential metabolites between the treatment combined MNC-168 and anti-PD1 versus anti-PD1 single treatment. The differential metabolites were identified with negative (a) and positive (b) property of metabolite by LC-MS.


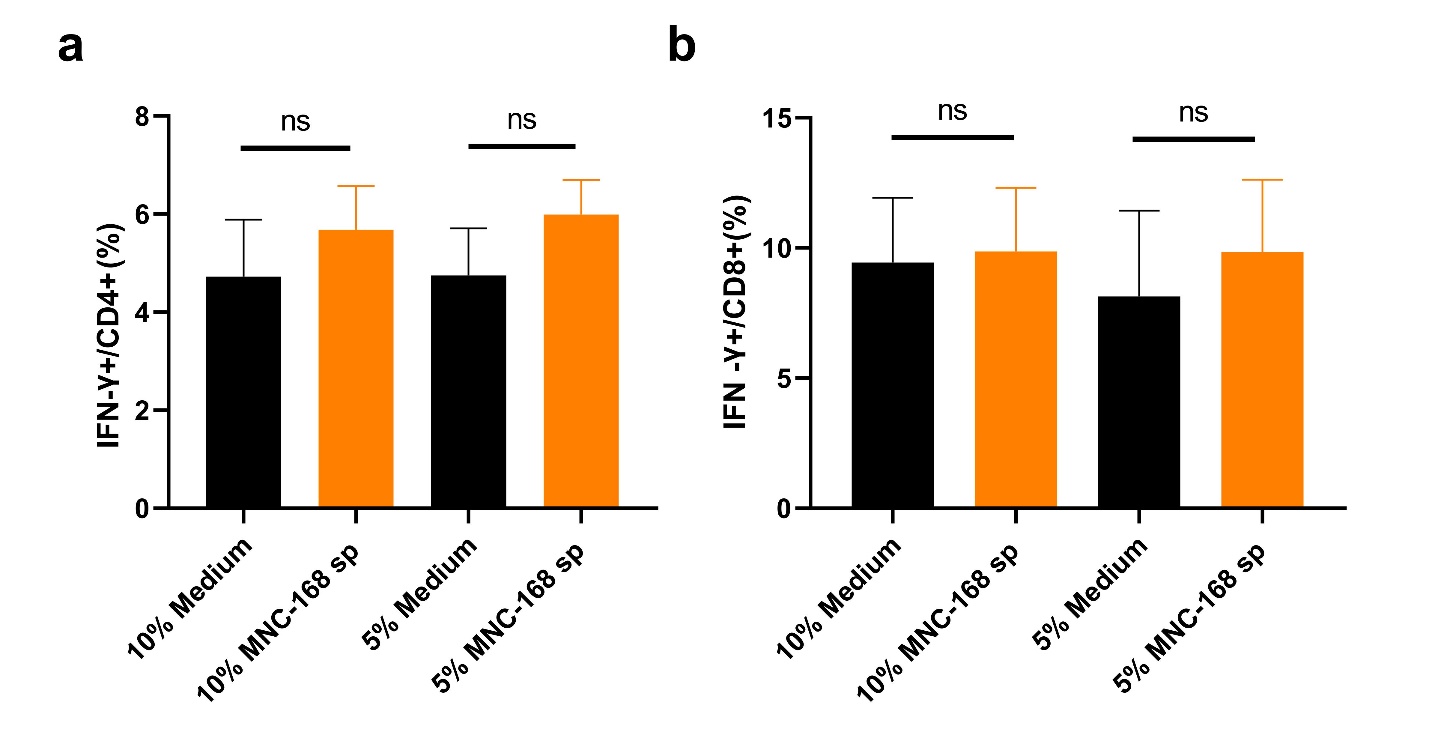


Figure. S7.

Evaluate the effect of MNC-168 on T cell activation. T cells were differentiated from Human PBMCs and co-cultured with MNC-168 supernatant, IFN-γexpression of CD 4^+^ (a) and CD8^+^ (b) T cell were detected by flow cytometry.


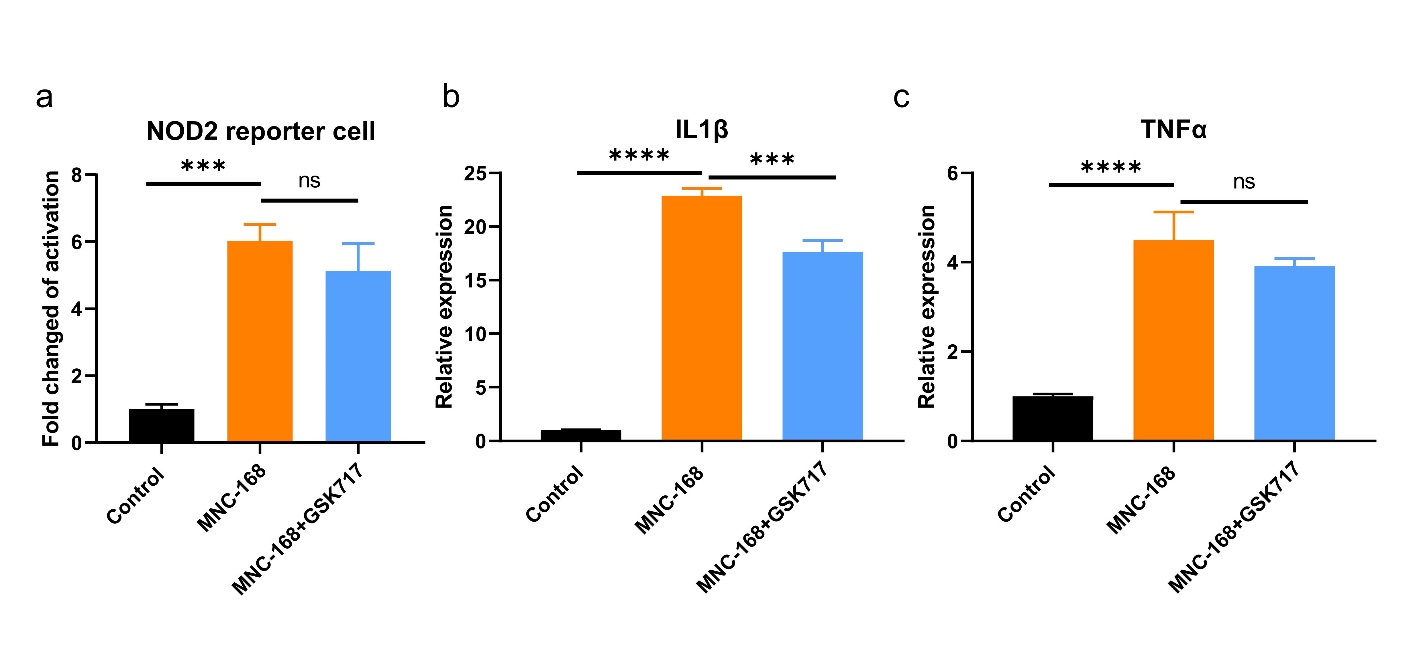


Figure. S8.

The regulation of MNC-168 may not be solely reliant on NOD2 signaling. a. Fold change of NFkB activity in THP-1 reporter cells stimulated with supernatant of MNC-168 (1:10 in volume) in the presence or absence of the NOD2 inhibitor GSK717 (10 µM), bacterial culture medium MM01 was used as control. b and c**.** The mRNA expression levels of IL-1β and TNF-α were assessed in THP-1 reporter cells using RT-PCR following the indicated treatments. For all graphs, n=3, data are shown as the mean ± s.e.m.; **P* < 0.05, ***P* < 0.005, ****P* < 0.0005, *****P* < 0.0001, NS, not significant. *P* values were calculated using one-way ANOVA with Tukey’s test for multiple comparison.


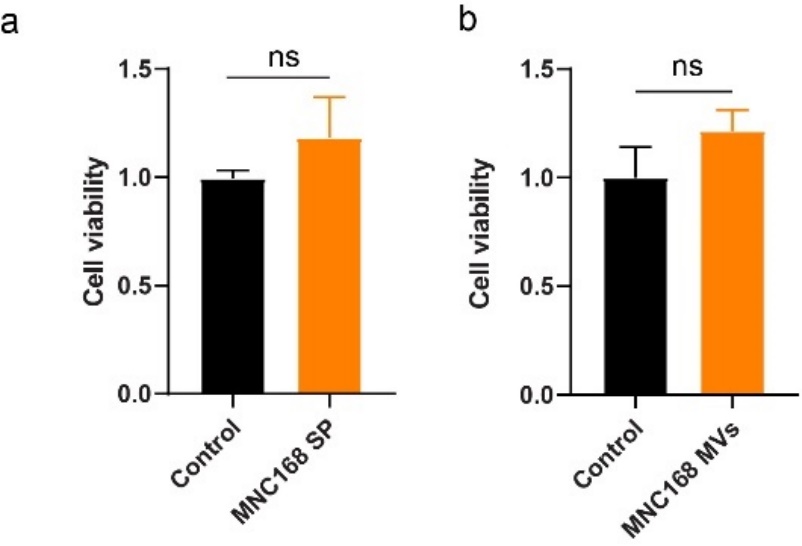


**Figure. S9.**

Cell viability assay for the influence of cell growth under MNC-168 supernatant (a) or MVs (b) treatment.


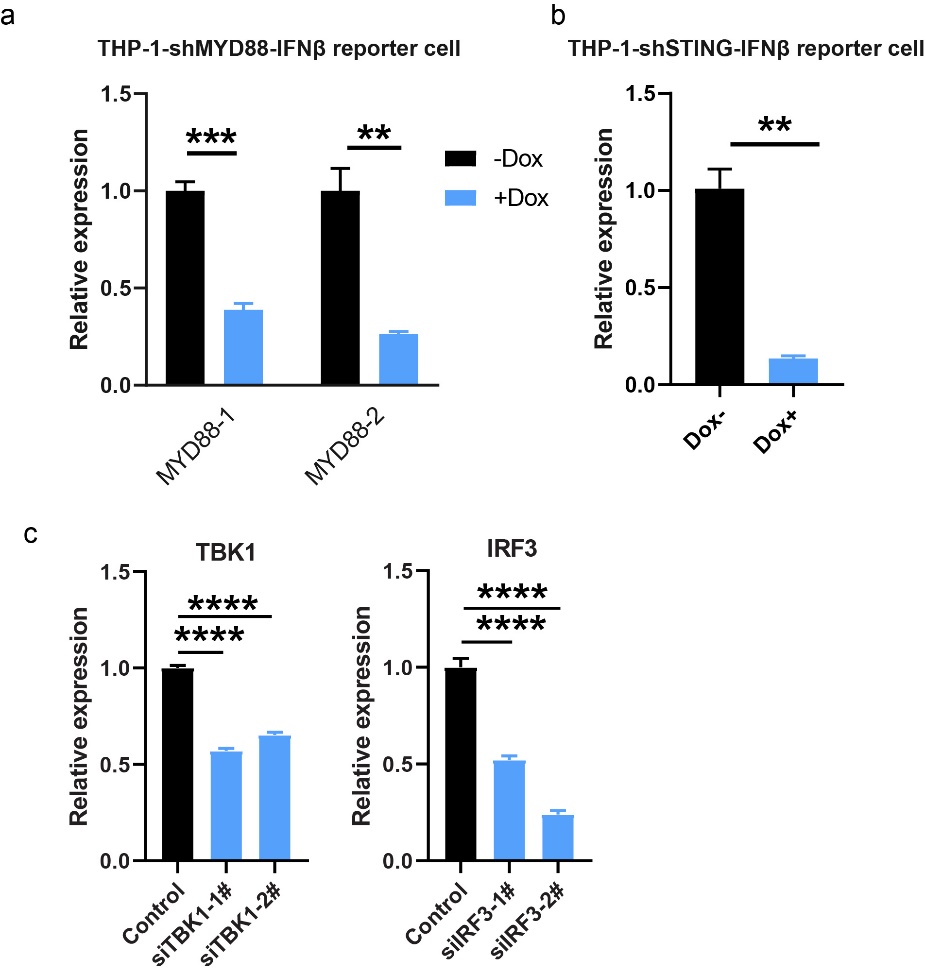


Figure. S10.

Evaluation of knockdown efficiency of cells. a. MYD88 expression was detected by RT-PCR under Dox induced knockdown in THP-1-shMYD88-IFNβ reporter cell. b. STING expression was detected by RT-PCR under Dox induced knockdown in THP-1-shSTING-IFNβ reporter cell. c. The expressions of TBK1 and IRF3 of THP-1- IFNβ reporter cell were detected by RT-PCR which knockdown with two siRNA fragments for 24 hours.


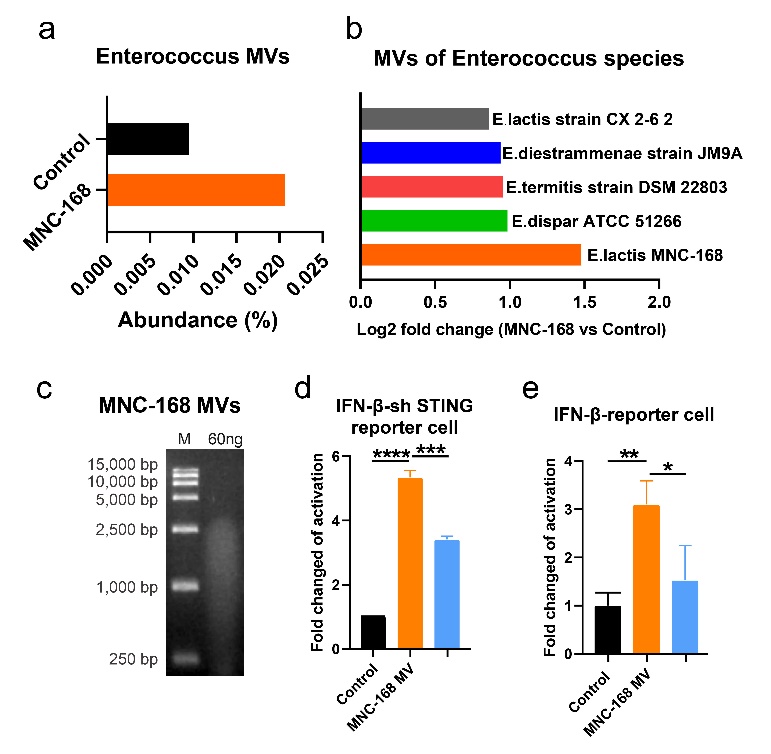


**Figure. S11.**

MNC-168 MVs induce STING pathway activation. a. Comparison of the abundance of Enterococcus genus MVs in faeces between control and MNC-168 treated. b. The fold changes of different Enterococcus species MVs in faeces in comparison between control and MNC-168 treated. c. The DNA of MNC-168 MVs was detected through agarose gel electrophoresis. d and e. Evaluation of IFN-β activity was performed using THP-shSTING-IFN-β-reporter cells treated with MNC-168 MVs (2.5×106 particles) under Dox-induced STING knockdown (d), in the presence or absence of the STING inhibitor H-151 (10 µM) (e).


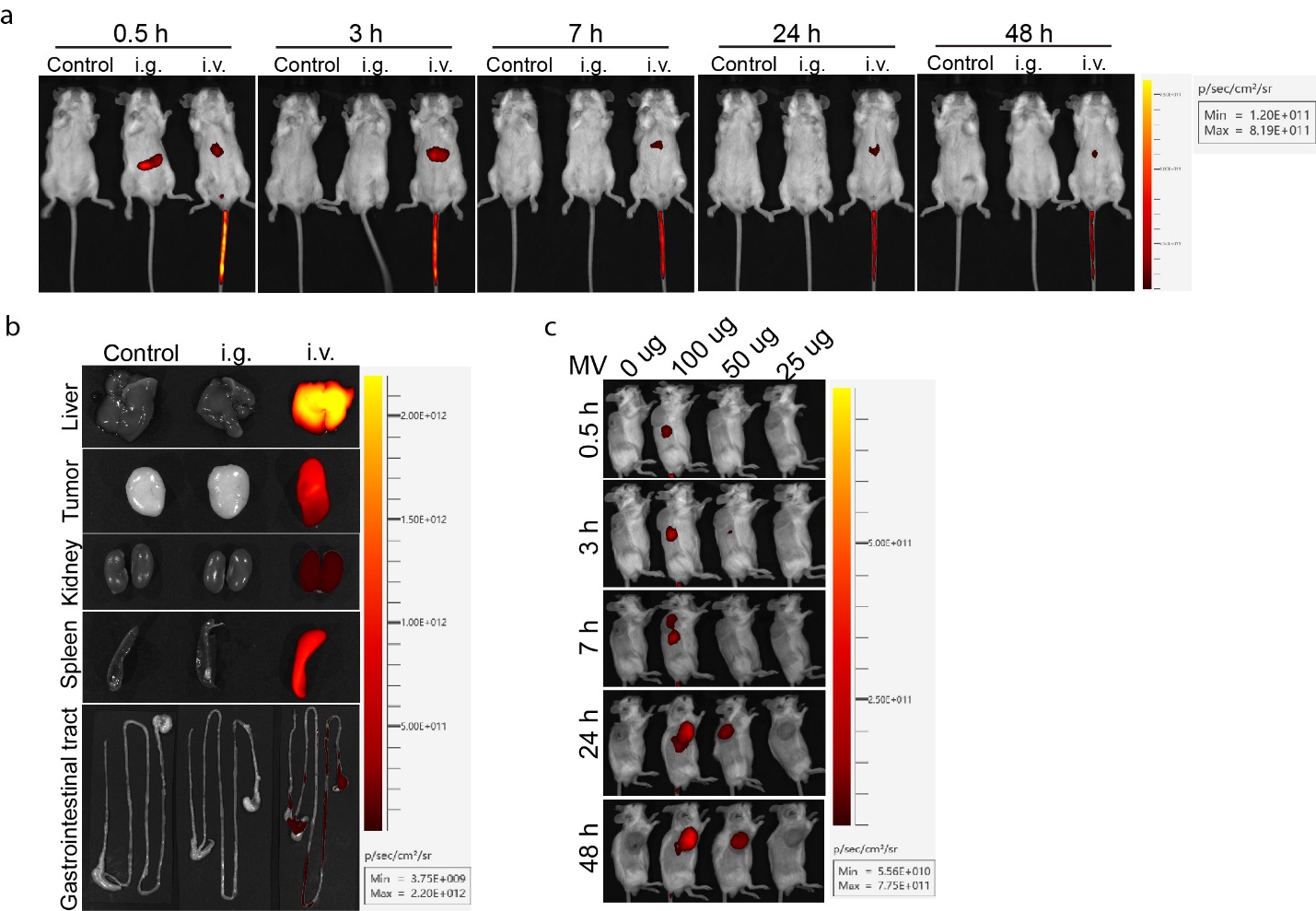


**Figure S12**

In vivo tracking of MNC-168 MVs. a. Images of the MVs tracking of MNC-168 MVs indicated timepoint. DiR labeled MNC-168 MVs (200 ug) were gavaged or int intravenous injected to the CT-26 bearing mouse and detected with indicated timepoint. b. Images of fluorescence intensity of different organ dissection post 48 hours treatment. c. Images of different doses of MVs in time-course experiment.


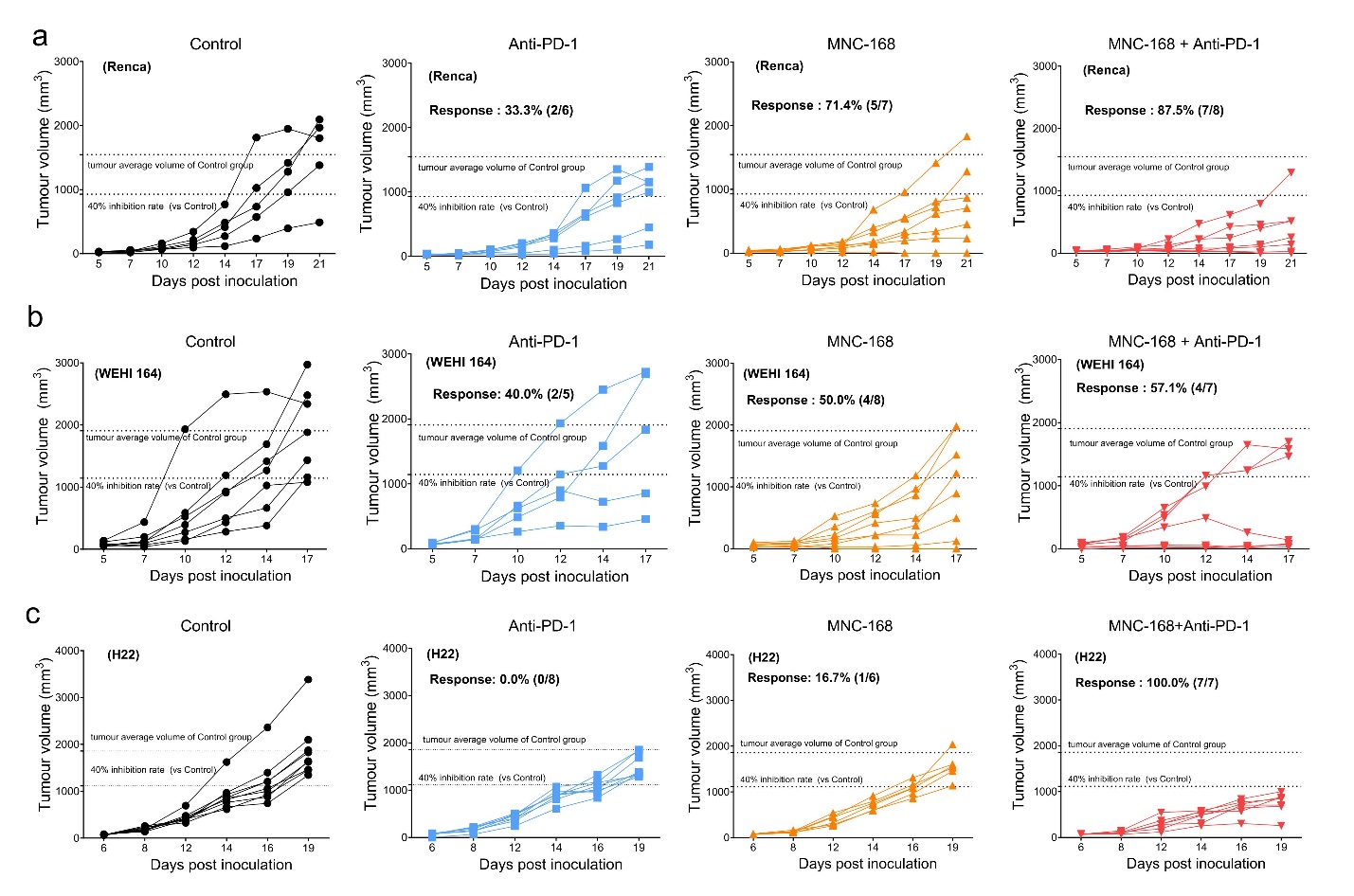


Figure. S13.

MNC-168 improved the responsive rate of anti-PD-1 treatment in refractory tumor. a-c. Growth curve of individual tumor with indicated treatment and the therapy responsive rate analysis. Tumor inhibition rate when compared with control group, equal or greater than 40%, is considered as response. a, Renca, renal cell carcinoma model; b, WEHI 164, fibrosarcoma model; c, H22, hepatocellular carcinoma model.


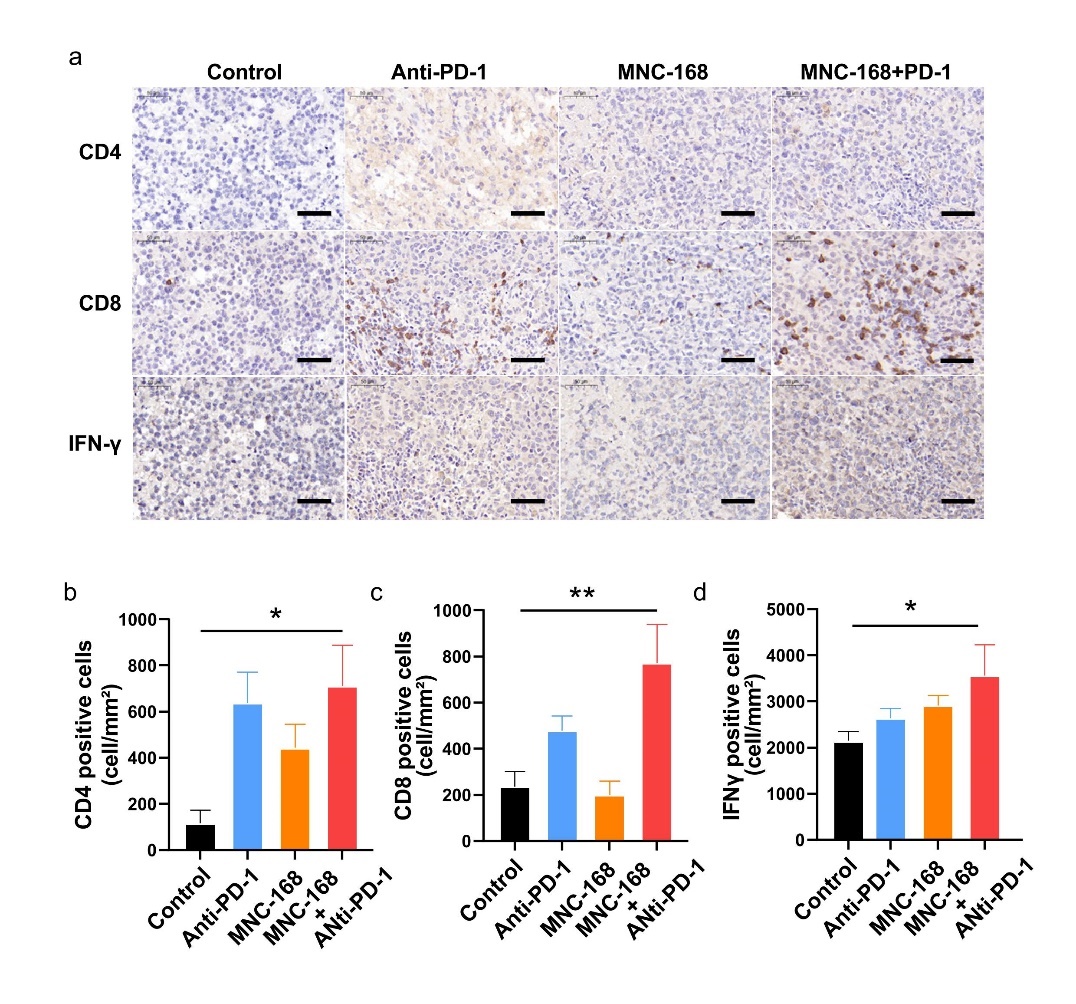


Figure. S14.

MNC-168 enhanced intratumoral T cell activation of murine H22 hepatocellular carcinoma cell model. a. Representative immunohistochemistry images of effect T in H22 tumor with MNC-168 administration with or without anti-PD-1. Tumor tissue slices with were stained with antibody of CD4, CD8, and IFN-γ. b-c. Quantitative analysis of the number of CD4, CD8, and IFN-γ positive cells in an area of the slide. Five random high power field images were used for statistical analysis. For all graphs, data are shown as the mean ± s.e.m.; **P* < 0.05, ***P* < 0.005, ****P* < 0.0005, *****P* < 0.0001, NS, not significant. P values were calculated using one way *ANOVA* with Tukey’s test for multiple comparison.


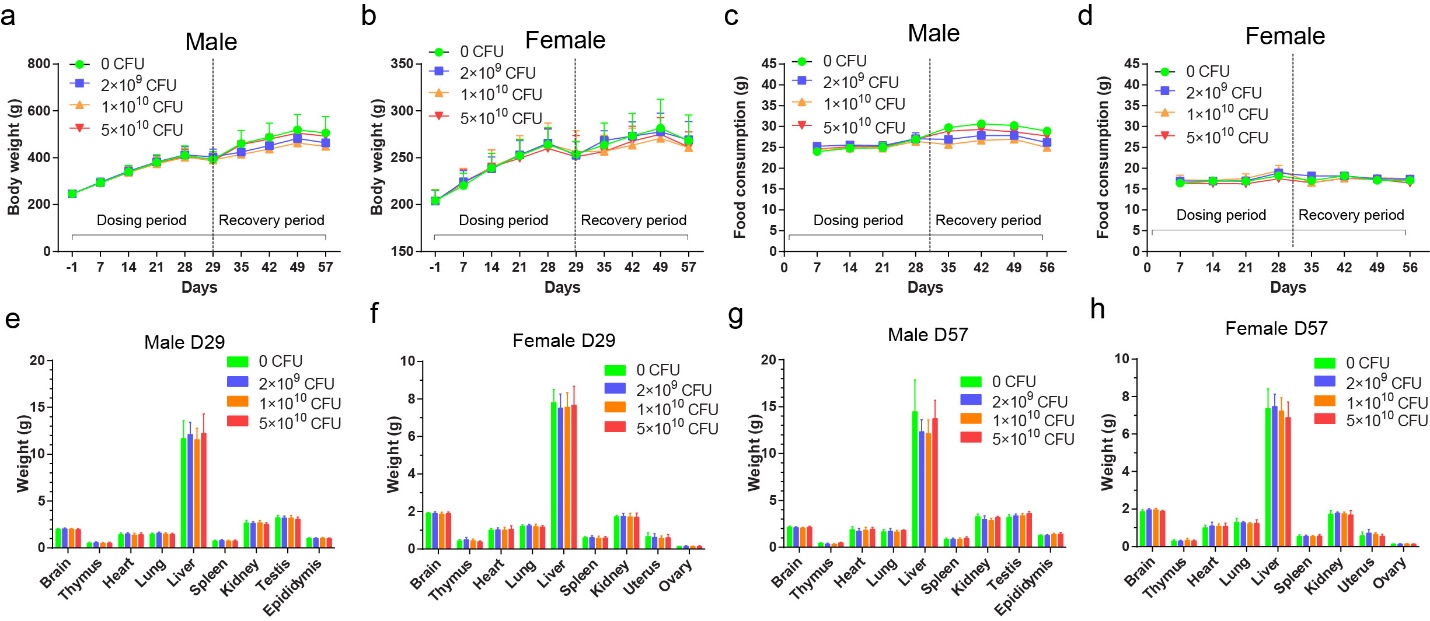


**Figure. S15.**

Toxicity study of MNC-168 in Sprague Dawley (SD) rats. a and b. Body weight growth curve of male or female rats for 8 weeks toxicity evaluation, the first 4 weeks are the peri-administration period, and the following 4 weeks are the recovery period. c and d. Food consumption of male or female rats. d and f. The weight of the organ at the autopsy on day 29. g and f. The weight of the organ at the autopsy on day D57

Supplementary files

**Supplementary file 1.**

Bacterial species enrichment analysis of melanoma patients with response or non-response to immunotherapy

**Supplementary file 2.**

Bacterial species enrichment analysis of NSCLC and RCC patients with response or non-response to immunotherapy

**Supplementary file 3.**

Cytokines detection of supernatant from MNC-168 and PBMC or THP-1 coculture

**Supplementary file 4.**

Identification of metabolites from the cecal contents

**Supplementary file 5.**
Differential expressed genes of MNC-168 combine anti-PD-1 vs anti-PD-1

**Supplementary file 6.**

GSEA of DEGs

**Supplementary file 7.**

Data summary of hematology and coagulation the toxicity study.
